# The Conditioned Environmental Center‐Periphery Hypothesis of Biogeography: Statistical Evidence From Tree Species

**DOI:** 10.1002/ece3.70934

**Published:** 2025-03-16

**Authors:** Pablo Antúnez, Martin Ricker

**Affiliations:** ^1^ División de Estudios de Postgrado Universidad de la Sierra Juárez Ixtlán de Juárez Oaxaca Mexico; ^2^ Departamento de Botánica, Instituto de Biología Universidad Nacional Autónoma de México (UNAM) Ciudad de México Alcaldía Coyoacán Mexico

**Keywords:** abundant‐center hypothesis, centroid niche hypothesis, environmental gradient, geographical distribution, highest‐probability interval, logistic regression, statistical probability density function

## Abstract

It has been discussed for decades whether species occur most frequently at their geographic center, and more recently at their environmental niches' center. The aim here is to analyze for each environmental gradient separately the ecological niche of 12 Mexican tree species and 16 abiotic environmental gradients, in the form of statistical probability density functions. Is a symmetrically positioned center always possible by searching for additional data? For each species‐variable combination, the occurrences along an environmental gradient were grouped in histograms. Logistic regression was used to fit a polynomial equation, whose degree depended on the number of significantly different bins. A highest‐probability interval on the gradient was determined, where 25% of the individuals were found with the highest probability. The relative distance from the center (midpoint) of the variable's range was calculated, and the feasibility of expanding the encountered interval on the environmental gradient for symmetry was analyzed. For 183 species‐variable combinations, in only 22 cases (12.0%) did the highest‐probability intervals include the midpoint of the environmental gradient. Furthermore, for 55% of the species‐variable combinations, the truncation of the environmental gradients for species makes it impossible to expand the measured range with additional data for the shorter tail. For example, precipitation cannot be negative. This truncation frequently causes asymmetry around the highest‐probability intervals. In those cases, the classical environmental center‐periphery hypothesis turns out to be wrong, whereas in the remaining cases it could apply. This has implications for biogeographical assumptions, such as where to identify the best areas for conservation or how to predict the effects of climate change. We propose a new conditioned environmental center‐periphery hypothesis: “On an environmental gradient, a given species is able to cover a certain range. For environmental gradients, where natural truncation of the environmental gradient is not limiting, the highest probability of occurrence is found away from the range's endpoints, tending towards its midpoint.”

## Graphical abstract

We propose the following new environmental center‐periphery hypothesis that is conditioned: “On an environmental gradient, a given species is able to cover a certain range. For environmental gradients, where natural truncation of the environmental gradient is not limiting, the highest probability of occurrence is found away from the range's endpoints, tending towards its midpoint.”

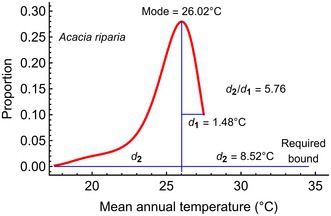

## 1 Introduction

It has been discussed for decades whether species occur most frequently or abundantly at their geographic center, and more recently, at their environmental niches' center (Dallas et al. 2017; Soberón et al. 2018; Fristoe et al. 2023). Species, including tree species, are adapted to environmental conditions (Flood and Hancock 2017); consequently, species are expected to have preferred points or intervals on unidimensional environmental gradients (Toledo et al. 2012). According to the geographical *center‐periphery hypothesis*, the probability of occurrence of a species is highest at the center of its range and decreases towards the edges, where conditions are unfavorable (Ntuli et al. 2020: 1). When abundance (or “density” of individuals) is the focus, the hypothesis is generally called the *abundant‐center hypothesis*, but we take the more general term of “center‐periphery hypothesis,” as described in Pironon et al. (2017; 1878), because our focus is on the probability of occurrence. Here we analyze the frequency of the occurrence of a species at different geographic sites, independently if the species is abundant (with many individuals) or not at any particular site.

The early idea was to look at the center of a geographic distribution, which can be referred to as the *geographical center‐periphery hypothesis* (Sagarin and Gaines 2002). Over a geographic range; however, site conditions may change considerably, some being more suitable and others less suitable for a given species (Dallas et al. 2020). Pironon et al. (2017: 1900) concluded that “Ecological conditions do not systematically follow geographic gradients”. Therefore, an improved concept analyzes environmental gradients (Martínez‐Meyer et al. 2013), which leads to the *environmental center‐periphery hypothesis* that is treated here (also called “centroid niche hypothesis”). The environmental center‐periphery hypothesis implies that the preferred position of a species is not found at a geographical center (or only by coincidence), but rather that multiple sites, possibly dispersed throughout a species' geographic distribution, can represent the preferred environmental conditions. On the other hand, the extreme ends of an environmental gradient could be geographically close, but distant from the periphery of the geographic distribution.

Here we analyzed data from 12 Mexican tree species and 16 environmental gradients. The data were used to develop and illustrate our statistical approach to analyze species occurrence on environmental gradients, testing the environmental center‐periphery hypothesis. We will focus in our analysis on the realized niches, rather than the fundamental niches, because this is what can be analyzed with field data and because of its direct relevance for applications. The potentially wider fundamental niche would require experiments on environmental gradients to verify the possibility of existence beyond the extremes that are observable in nature, and the discussion of why these more extreme parameters are not realized in nature.

## 2 Materials and Methods

### 2.1 Species and Data of Their Geographical Distributions

**FIGURE A1:**
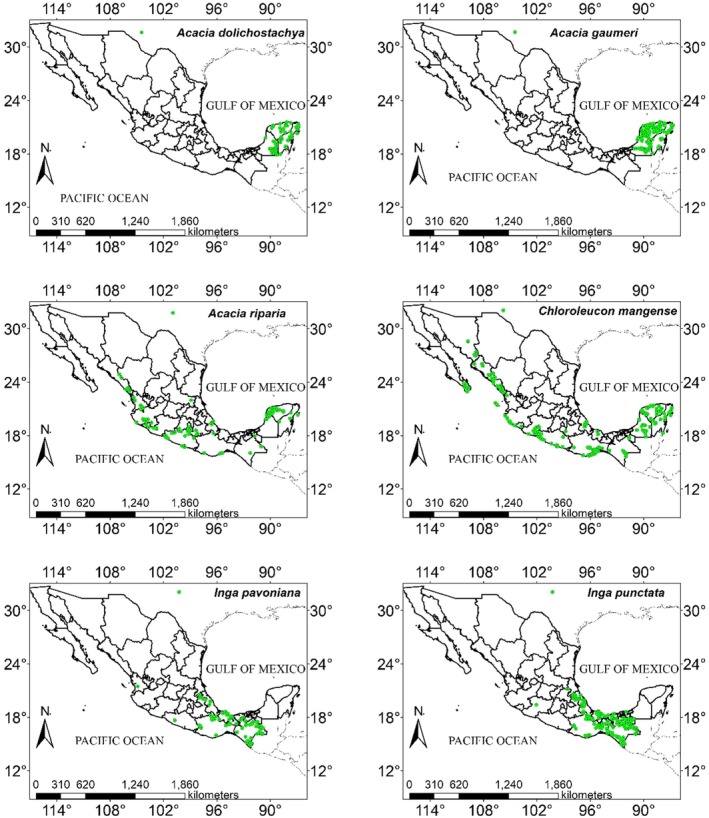
Maps of the species' distribution in Mexico.

**FIGURE A2:**
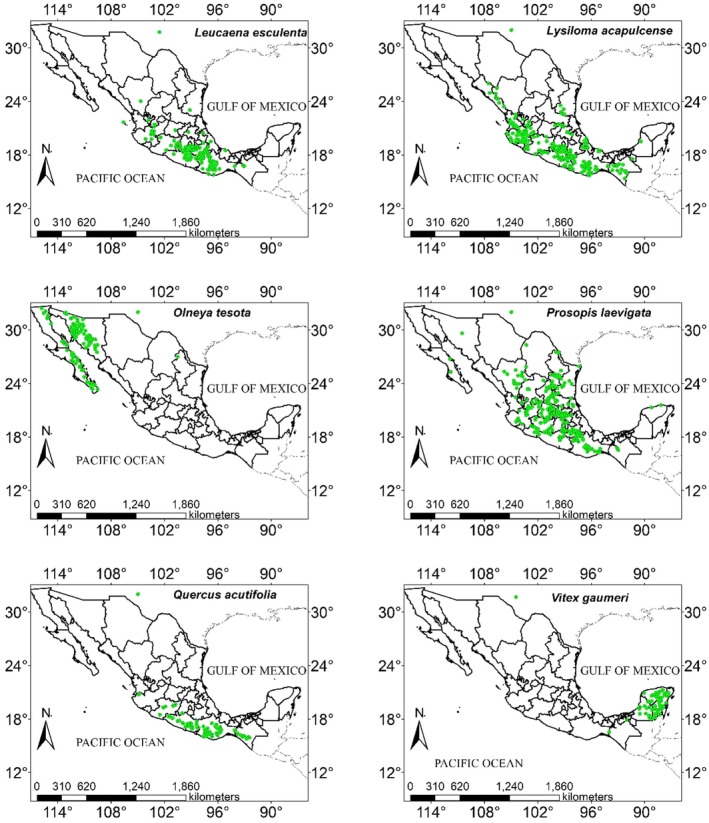
Maps of the species' distribution in Mexico (continued).

We studied 12 tree species that grow naturally in different forested regions of Mexico (Table 1; Figures A1 and A2 in Appendix A). The interest was in species with reliable taxonomic identification at a sufficiently high number of accurately georeferenced collection sites. The data of nine species of the family Fabaceae in Table 1 were taken from the database of an atlas of 174 Mexican tree species of the mimosoide clade in the legume family (Hernández et al. 2020). The data of the other three tree species ( *Olneya tesota* , *Quercus acutifolia*, and *Vitex gaumeri*) were taken from the database of Mexico's national forest inventory (2013–2016), compiled at the National Herbarium of Mexico (MEXU) from specimens sent from the field for identification at the MEXU (Ricker et al. 2015). The predominance of the family Fabaceae is due to the available data and the wide range of environmental conditions, in which many species are found. *Quercus acutifolia* and *Vitex gaumeri* were included in order to not only use a single plant family. The herbarium specimens had been identified and/or verified by taxonomic specialists at the MEXU, located in the Instituto de Biología (UNAM). Maps for each species with the collection sites are shown in Figures A1 and A2 of Appendix A, with the geographic coordinates given in Supporting Information S1a with the raw data (available at https://doi.org/10.17632/3pyh9jpgzg.1). For each specimen, the data in S1a is complete for all variables, and for each variable, the data are complete for all specimens. This avoids a possible bias from including specimens only for variables with a specific situation about the preferred location on a gradient.

**TABLE 1:** List of the 12 studied species with the number of collection sites, distance statistics among sites, and the corresponding ranges of three well‐known environmental variables.

Species^a^	Number of collection sites	Min to max, mean & median distance (km)^b^	Elevation (m)	Mean annual temperature (°C)	Mean annual precipitation (mm)	Presence in countries other than Mexico
*Acacia dolichostachya* S.F. Blake	95	1.1–642, 203 & 211	1–396	24.9–26.5	658–1488	Belize, Guatemala
*Acacia gaumeri* S.F. Blake	140	1.0–912, 187 & 194	1–304	25.3–26.7	638–1383	None
*Acacia riparia* Kunth	106	1.0–2120, 777 & 795	2–1642	17.8–28.0	568–2605	Central and South America, Caribbean
*Chloroleucon mangense* (Jacq.) Britton & Rose	239	1.0–2507, 882 & 958	0–1217	19.5–28.1	231–2422	Central and South America, Caribbean
*Inga pavoniana* Benth	95	1.0–1588, 329 & 364	3–1825	19.2–27.9	900–4390	Central and South America, Caribbean
*Inga punctata* Willd	213	1.0–1247, 319 & 344	3–1591	17.3–27.2	917–4328	Central and South America, Caribbean
*Leucaena esculenta* (DC.) Benth	176	1.2–1529, 241 & 305	40–2450	13.6–27.9	304–3789	Originally none, but nowadays widely cultivated in the tropics
*Lysiloma acapulcense* (Kunth) Benth	287	1.2–1978, 473 & 526	7–2385	14.9–28.1	505–3833	Central America (Guatemala to Nicaragua)
*Olneya tesota* A. Gray	109	2.4–1591, 299 & 349	12–797	18.7–24.5	72–621	Southwestern United States
*Prosopis laevigata* (Humb. & Bonpl. ex Willd.) M.C. Johnst	321	1.2–2473, 423 & 479	3–2608	14.1–29.5	108–1485	Southwestern United States, Bolivia and Argentina
*Quercus acutifolia* Née	85	4.9–1420, 331 & 374	732–2794	13.0–24.8	642–3289	Belize, Guatemala, El Salvador
*Vitex gaumeri* Greenm	70	5.0–854, 170 & 189	1–795	23.4–26.6	788–2243	Central America and Colombia

^a^ All but two species belong to the family Fabaceae; *Quercus* belongs to Fagaceae, and *Vitex* to Lamiaceae.

^b^ Min = minimum distance, max = maximum, the number of data is $\left(n^{2}-n\right)/2$, where n is the number of collection sites from the previous column.

We employed a total of 1936 georeferenced points, where herbarium samples of the 12 species had been collected. All available specimens on distinct sites were used for each species from the projects. There were only 19 specimens (1.0% of 1936) that shared some of the 1917 sites, and none were of the same species. Repeated collections of the same species from the same site would result in a spurious increase of occurrence under the conditions of that particular site. The goal was to detect occurrence under varying abiotic site conditions (but not abundance on sites). The mean distance among collection sites of the same species was 170–882 km, and the minimum distance was 1.0 km (Table 1). The table further shows that depending on the species, the number of collection sites was 70–321. The elevation above sea level of the collection sites for all species varied from 0 to 2794 m, the mean annual temperature from 13.0°C to 29.5°C, and the mean annual precipitation from 72 to 4390. The last column shows that all but one species are also found outside Mexico, although we did not include data from other countries. The complete raw (input) data can be consulted in Supporting Information S1a.

### 2.2 Data of Environmental Variables

For each collection site, we determined the corresponding values of 16 independent variables to characterize aspects of climate, soil, and physiography. The summary table in Appendix B presents a short explanation and the overall ranges for each variable. Since there were generally no meteorological stations in the immediate neighborhood of the collection sites, values for the climate variables were obtained from spatial climate models, developed with the software ANUSPLIN 4.3 (Hutchinson and Xu 2004). The spatial resolution for all environmental variables was 0.0083 decimal degrees, or about 1 km. The climatic layers were obtained from https://forest.moscowfsl.wsu.edu/climate/details.php. They are based on the available daily records from 1961 to 1990 of average temperature, average minimum and maximum temperature, and total rainfall of the network of over 4000 local meteorological stations, distributed throughout Mexico and surrounding regions, including the southern United States, Cuba, Belize, and Guatemala (Rehfeldt 2006; Rehfeldt et al. 2006; Sáenz‐Romero et al. 2010; Crookston 2014). The data of the terrains' slopes are based on digital elevation models, available on the website of the National Institute of Statistics and Geography of Mexico (INEGI) (http://en.www.inegi.org.mx/app/mapas/). The pH value and cation exchange capacity of the soil were acquired from a soil mapping system, based on a global compilation of soil profiles, with a 1 × 1 km spatial resolution (https://www.isric.org/explore/soilgrids) (Hengl et al. 2017). This system predicts general soil properties using global covariates and globally fitted models, based on more than 6 million soil records around the world (Hengl et al. 2014, 2017; Shangguan et al. 2017). Whereas such data includes much uncertainty for a specific site, it may give an idea for the general soil characteristics of a region.

### 2.3 Moving Away From the Midpoint on a Single Environmental Gradient Versus in a Hyperdimensional Space

The center for a single environmental gradient of an ecological niche is the midpoint of the variables' ranges. With several variables, the niche center is the centroid in a multidimensional space, defined by the ranges of all variables for a given species. Ecologists have focused on analyzing the environmental center‐periphery hypothesis with multidimensional niche model, with a centroid and a distance in mutidimensional variable‐space (Martínez‐Meyer et al. 2013: 3, Yañez‐Arenas et al. 2014: 36, Osorio‐Olvera et al. 2016: 1082, Osorio‐Olvera et al. 2020: 557). This approach, however, represents an unnecessary complication. Moving away from the midpoint in a single dimension causes moving away from the centroid of an *n*‐dimensional hypervolume. Mathematically, consider the centroid to be at the end of a vector with coordinates $c_{1},c_{2},\ldots ,c_{n}$ in *n*‐dimensional space. The linear distance between two vectors is calculated with the Euclidean distance. The Euclidean distance $d_{E}$ of the centroid from the origin is

$$d_{E}=\sqrt{\sum_{i=1}^{n}{\left(c_{i}-0\right)}^{2}}=\sqrt{\sum_{i=1}^{n}c_{i}^{2}},$$

where i refers to the $i^{\mathrm{th}}$ dimension. Now, consider that the preferred position in the hyperdimensional niche is changed in dimension i by distances $d_{i}$ between the centroid at $c_{i}$ and the elsewhere preferred position at $c_{i}+d_{i}.$ The affected environmental gradients cause the Euclidean distance $d_{E}$ of the preferred position from the origin to become

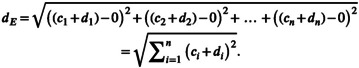




The distance between the centroid and the preferred position becomes

$$\begin{array}{c} d_{E}=\sqrt{{\left(\left(c_{1}+d_{1}\right)-c_{1}\right)}^{2}+{\left(\left(c_{2}+d_{2}\right)-c_{2}\right)}^{2}+\ldots +{\left(\left(c_{n}+d_{n}\right)-c_{n}\right)}^{2}}\\ =\sqrt{\sum_{i=1}^{n}d_{i}^{2}}. \end{array}$$

With only one environmental gradient presenting a distance $d_{i}$ away from its center, the Euclidean distance from the origin to the position in multidimensional space becomes

$$d_{E}=\sqrt{c_{1}^{2}+\ldots +{\left(d_{j}-c_{j}\right)}^{2}+\ldots +c_{n}^{2}},$$

and the distance from the centroid becomes $\sqrt{d_{j}^{2}}=d_{j}$. One sees that a distance of a single variable changes directly the Euclidean distance from the centroid in multidimensional space. On the other hand, to analyze if the preferred position on environmental gradients is at the niche's centroid or not, we do not have to focus on calculating $d_{E}$ in multidimensional space. It is sufficient to focus on unidimensional distances $d_{i},$ because we know that a single $d_{i}>0$ causes $d_{E}$ to change, and the hyperdimensional position to move away from the original centroid. The instantaneous change of $d_{E}$ in this case can be calculated as

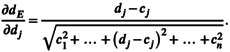




Consequently, one can analyze the whole issue of a preferred midpoint largely with a unidimensional model (as in Canham and Thomas 2010), and think of multiple dimensions in terms of an additive effect among several single dimensions. Analytically, it is much easier to analyze single dimensions: The inspection, interpretation, and discussion can be carried out one variable at a time. Furthermore, one works with deviation to the left or right from the midpoint, which is helpful for detecting the highest frequency at a specific point. This feature is lost by calculating the Euclidean distance (or another absolute distance function) in multidimensional space.

### 2.4 Correlation Analysis Among Environmental Variables

One question that results in the analysis of many environmental variables separately is if they nevertheless “move together” to some extent. Thinking of a hypervolume niche around a centroid, correlations can affect the relationship among preferred positions on the considered environmental gradients. Assume that $\Delta d_{2}=\Delta d_{1}\cdot r_{12}$, where Δ refers to a change of the distance and $r_{12}$ to the correlation coefficient between $d_{1}$ and $d_{2}$, and that only these two distances to the centroid exist. The Euclidean distance between the centroid and the preferred position is $d_{E}=\sqrt{d_{1}^{2}+d_{2}^{2}}$. A change of $d_{1}$ causes it to change to

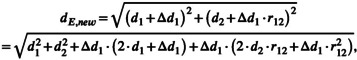

which includes the interaction term $\Delta d_{1}\cdot \left(2\cdot d_{2}\cdot r_{12}+\Delta d_{1}\cdot r_{12}^{2}\right)$ between the two variables (distances), in comparison with the case without correlation. However, whereas the preferred position on an environmental gradient in a unidimensional analysis could be influenced by other variables, here we neither calculate the overall distance to the centroid nor are we analyzing change. Our empirical analysis of the preferred position on an environmental gradient detects the *status quo*, *id est*, the position under the current state of other variables, and that is what we are interested in. Correlations would have an important role in the analysis of the effect of changing preferred positions on one environmental gradient on the change of the preferred position on other environmental gradients. In our analysis, in contrast, some variables might simply not provide much additional information, as there could be only a few variable groups with highly correlated variables. It could result in spurious conclusions about the number or proportion of variables that support the environmental center‐periphery hypothesis, even though, other than that, there is no direct problem for our analyses.

To determine the frequency of high correlations, we calculated pairwise $\left(16^{2}-16\right)/2=120$ Pearson correlation coefficients among variables (Sokal and Rohlf 2012; chapter 15), with the data given in supplementary information S1a. There were no missing values. The calculations were carried out with *Mathematica*'s function “Correlation.” Statistical significance of the coefficients $\left(r\neq 0\right)$ was not tested, and thus no statistical assumptions about the data's distributions were assumed.

### 2.5 Estimation of Species' Occurrence on an Environmental Gradient With Polynomial Logistic Regression

**FIGURE 1:**
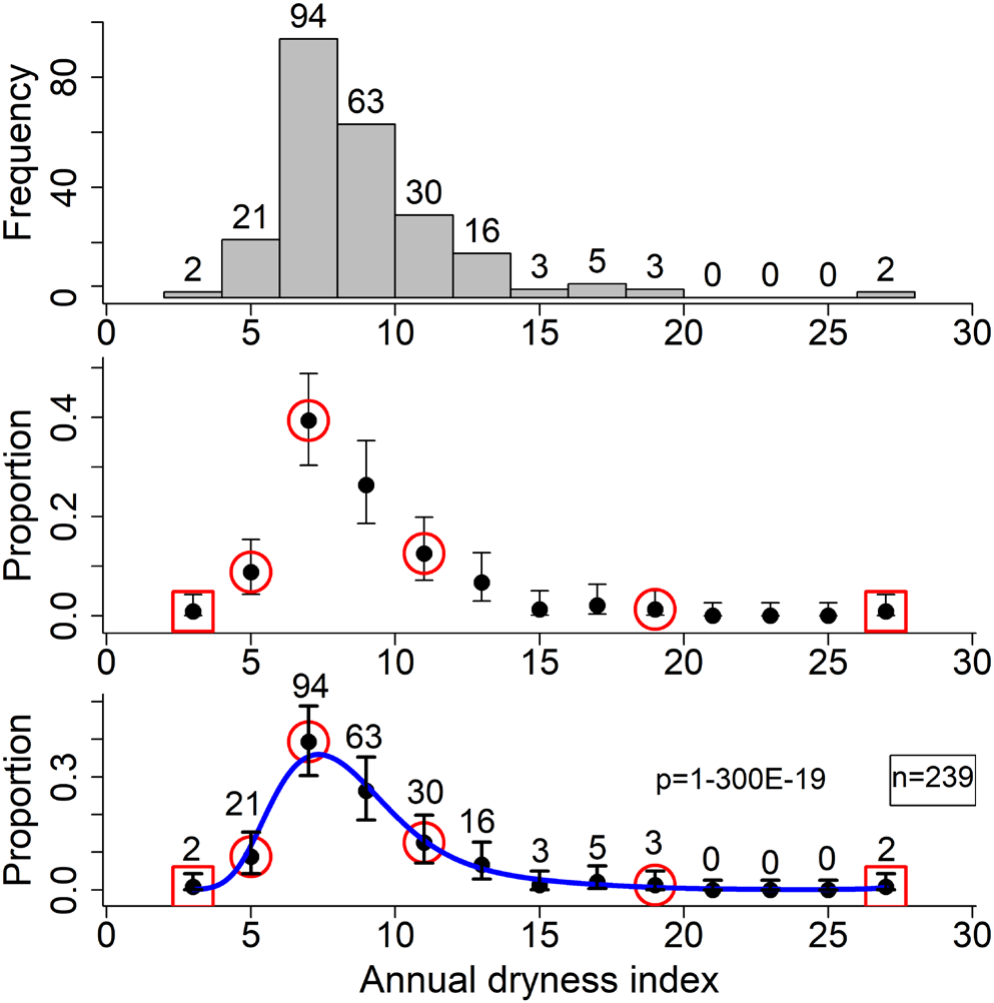
Example of one of 183 analyses for the species‐variable combinations. Here, the species is *Chloroleucon mangense*, and the variable is the annual dryness index. *Above*: A histogram of 239 data points of annual dryness index for the species is generated according to Sturges' (1926) rule. *Middle*: Absolute heights from the histogram were divided by the total number of occurrences (*n* = 239), resulting in proportions between 0 and 1 that can be interpreted as probabilities (for example, 94/239=0.39). Black filled circles correspond to the bins' relative height and midpoints, with 95% Clopper‐Pearson confidence intervals indicated as black intervals. The red empty circles show significantly different points, and the red empty squares the endpoints. With six marked points, the polynomial's degree for the logistic regression is chosen to be five. *Below*: The logistic‐regression line is added in blue.

We developed the following method to estimate the probability of a species' occurrence as a continuous function of a specific environmental variable for each combination of 12 species and 16 environmental variables. The objective was to determine for each species‐variable combination a small interval on an environmental gradient, where the species has the highest probability of occurrence. There were several steps involved. First, the data for the environmental gradient of each species‐variable combination was used to create a histogram that showed how many of the collected specimens fall into different intervals (bins) over the range of the environmental variable. The histogram's bin widths and thus the number of classes were computed with the R‐package “graphics.” Using Sturges' (1926) rule, the bin width is twice the interquartile range of the data, divided by the cubic root of the number of data points (Figure 1 top).

Second, by dividing the height of each bin (frequency) in a histogram of a given species and environmental variable by the overall number of specimens in the histogram, the frequencies were converted into proportions. Supposing that sampling was representative for the species and environmental variable, these relative frequencies could be interpreted as probabilities. Each bin's midpoint on the *x*‐axis was taken as the environmental parameter for the corresponding probability. In this way, the histogram was converted into a diagram of points that relate the probability of a species' occurrence with different environmental parameters. Confidence intervals were calculated, in order to compare probabilities, and estimate pairwise which ones could be considered significantly different (Figure 1 middle). Technically, Clopper‐Pearson confidence intervals for proportions between 0 and 1 were calculated for each probability point (Clopper and Pearson 1934; Agresti and Coull 1998; Fleiss et al. 2003: 25), using the R‐package “DescTools.” Conceptually, confidence intervals can be calculated for a single point in the case of a binomial distribution, when knowing the corresponding sample size, because the mean and the variance are mathematically dependent (in contrast to the normal distribution). Given the encountered range of an environmental variable, the confidence interval refers to the probability to find a certain number of occurrences on different sites over the environmental parameter's bin width. To calculate confidence intervals for multiple comparisons, the significance level α was adjusted for the number of groups (*k*) that were compared. As a conservative and easy way, we employed the Bonferroni correction (Miller 1981: 67; Sokal and Rohlf 2012: 239). For a family‐wide error rate of α=0.05, one adjusts $\alpha_{\mathrm{adj}.}=\alpha /k$, so that in the case of six comparisons $\alpha_{\mathrm{adj}.}=0.0083$. The resulting confidence intervals can be compared pairwise: Non‐overlapping simultaneous 95% confidence intervals (or technically 99.17% after Bonferroni correction) imply that a significance test between two means would detect a significant difference below the 5% significance level. The wider the gap, the lower the probability that the two means could belong to the same statistical population. Confidence intervals that overlap slightly (indicating “no” statistical difference) do not imply necessarily that a significance test would not detect a significant difference (Schenker and Gentleman 2001)^1^, but are nevertheless an intuitive way to present different levels of statistical differences among means.

Third, an omnibus (or “global”) chi‐squared test according to Fleiss et al. (2003: 188–189) was carried out to confirm differences among the proportions within a graph of a given species‐variable combination, using the R‐function “pchisq.” With 12 species and 16 variables, there are 192 possible species‐variable combinations. In nine cases, all pairwise comparisons in a graph had overlapping 95% confidence intervals; that is, significant differences could not be detected, and were eliminated. The most likely explanation for the non‐significance is that the sample size was too small for detection (and not that all true probabilities were equal). It is not expected that species are indifferent to a gradient over a wide range of any environmental variable. The nine cases reduced the number of species‐variable combinations from 192 to 183 (4.7% less).

Fourth, to derive a continuous function of the probabilities as a function of an environmental variable for a given species, logistic regression with a polynomial regression model was used (Sokal and Rohlf 2012; 780–793), employing the R‐package “mgcv”with the following regression model:

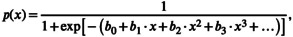

where $p\left(x\right)$ is interpreted as the probability of occurrence as a function of the environmental gradient. The minus sign in the denominator is maintained here from the original model $p\left(x\right)=1/\left(1+\exp\left[-f\left(x\right)\right]\right)$ for any function $f\left(x\right).$ The model results in a continuous regression line, with $0<p\left(x\right)<1.$ Note that we did not expect the coefficients of the logistic regression to be significantly different from zero. To judge significance, the confidence intervals of the probabilities had already been used, as well as the mentioned chi‐square test. The regression does not take into account the confidence intervals, and we used the regression rather as a convenient tool to derive a continuous function for the subsequent analyses (Figure 1 bottom). The degree of the polynomial in the logistic regression equation was determined by the application of four rules, with the objective to derive a continuous probability density function that reflects significantly different bin heights:

- In the graph of the points with the confidence intervals (Figure 1 middle), the highest point is marked. If there are ties, any one of the tied points can be chosen.
- On both sides of the highest point (if it is not an end point on the *x*‐axis), mark the next point on each side that clearly has no overlapping confidence interval (*id est*, it has a significantly different probability). Compared to this newly marked point, mark the next point without overlapping confidence intervals, and so on.
- When all significantly different points are marked, also mark the two end points on the *x*‐axis, if they are not marked already, even though their confidence intervals may not be statistically different from the neighboring ones. This rule avoids subsequent extrapolation of the regression toward the end points.
- In general, the number of polynomial terms (counting the *y*‐intercept *b*_0_ used for the logistic regression, is chosen to be equal to the number of marked points. The degree of the polynomial is thus one less than the number of marked points. After visual inspection, we used additionally one term less in three cases, and one more term in five cases (representing 4.4% of the 183 species‐variable combinations), to remedy a somewhat erratic behavior of the polynomial.

bottom). The degree of the polynomial in the logistic regression equation was determined by the application of four rules, with the objective to derive a continuous probability density function that reflects significantly different bin heights:

### 2.6 Estimation of the Highest‐Probability Intervals for Environmental Variables

**FIGURE 2:**
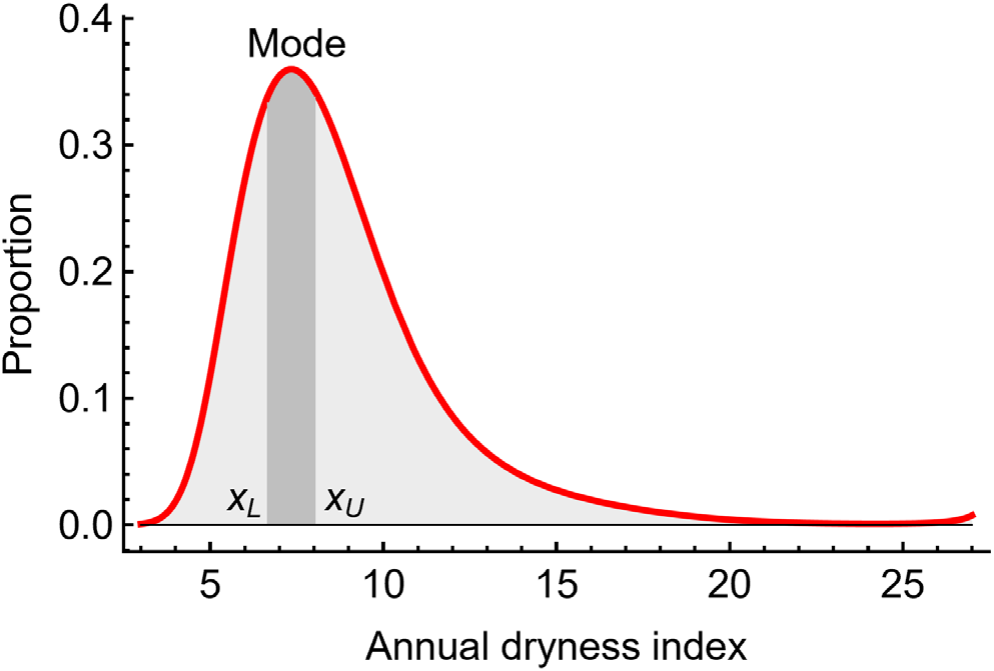
Highest‐probability interval from $x_{L}$ to $x_{U}$ in dark gray. The polynomial logistic regression equation for $p\left(x\right)$ from Figure 1 bottom is shown as a red line, with the area under the curve representing the integral. A quarter area of the total area is shown in dark gray around the mode. In cases, where the mode is on an endpoint, the quarter area was calculated only on one side of the mode.

We define the highest‐probability interval as the interval on an environmental variable of a given species, where 25% of the occurrences are found with highest probabilities (25% was chosen arbitrarily by us as a reasonable percentage). An example is shown in Figure 2. A relatively large interval provides a large margin of uncertainty for the presence of a peak. The area under the polynomial logistic regression line can be interpreted as a statistical probability density function, if it is standardized to sum up to 1 (Hogg and Tanis 1993: 117, 192). Subsequently, the 25% of the area with the highest probability density can be determined. Since a quarter area is a relative determination, the standardization to 1 can be skipped. Appendix C describes the algorithm for calculating $x_{L}$ and $x_{U}$, using the example from Figure 1 (“L” for “lower limit,” and “U” for “upper limit”). The procedure was carried out for each of the 183 species‐variable combinations.

### 2.7 Derivation of the Relative Distance of the Midpoint From the Nearest Highest‐Probability Limit for Each Variable

The midpoint $\left(x_{\mathrm{mid}}\right)$ of an environmental gradient represents the center of the niche for the variable *x*, and is defined as $x_{\mathrm{mid}}=\left(x_{\min}+x_{\max}\right)/2$, where $x_{\min}$ and $x_{\max}$ refer to the minimum and maximum values for a given species‐variable combination. For each environmental gradient of a given species, we calculated the relative distance $\left(\text{relDis}\right)$ of the niche's nearest highest‐probability limit from the midpoint. With $x_{L}$ and $x_{U}$ from the last section being the highest‐probability limits, we define the relative distance as the distance outside of the interval of $x_{L}$ to $x_{U}$, relative to the overall environmental range (100%). There are three cases for calculating relDis:

$$\begin{array}{l} x_{\mathrm{mid}}<x_{L}\Rightarrow \text{relDis}=\frac{x_{L}-x_{\mathrm{mid}}}{x_{\max}-x_{\min}}\cdot 100\%,\\ x_{L}\leq x_{\mathrm{mid}}\leq x_{U}\Rightarrow \text{relDis}=0,\\ x_{\mathrm{mid}}>x_{U}\Rightarrow \text{relDis}=\frac{x_{U}-x_{\mathrm{mid}}}{x_{\max}-x_{\min}}\cdot 100\%. \end{array}$$

With a relative distance of zero, the midpoint can be anywhere between the highest‐probability limits $x_{L}$ and $x_{U}$. Note that for $x_{L}<x_{\mathrm{mid}}$, the relative distance is negative. The mathematical domain of the relative distance is from −50% to 50%, which can be seen by substituting on the righthand‐side $x_{\mathrm{mid}}$ with $\left(x_{\min}+x_{\max}\right)/2$, $x_{L}$ with $x_{\min}$, and $x_{U}$ with $x_{\max}$.

## 3 Results

Figure 3 shows three examples with different shapes of the statistical distributions, resulting from the logistic regression for each species‐variable combination. Details of the regression equations are provided in the Supporting Information S1b, and all 183 graphs are shown in Figures D1, D2, D3, D4, D5, D6, D7, D8, D9, D10, D11, D12, D13, D14, D15, D16, D17, D18, D19, D20, D21, D22, D23, D24, D25, D26, D27, D28, D29, D30, D31, D32 of Appendix D. Rarely did we encounter an indication of two peaks, which could indicate either two varieties of the species or interaction among variables (and obviously not one midpoint center anymore). Typically, one peak was clearly dominating.

**FIGURE 3:**
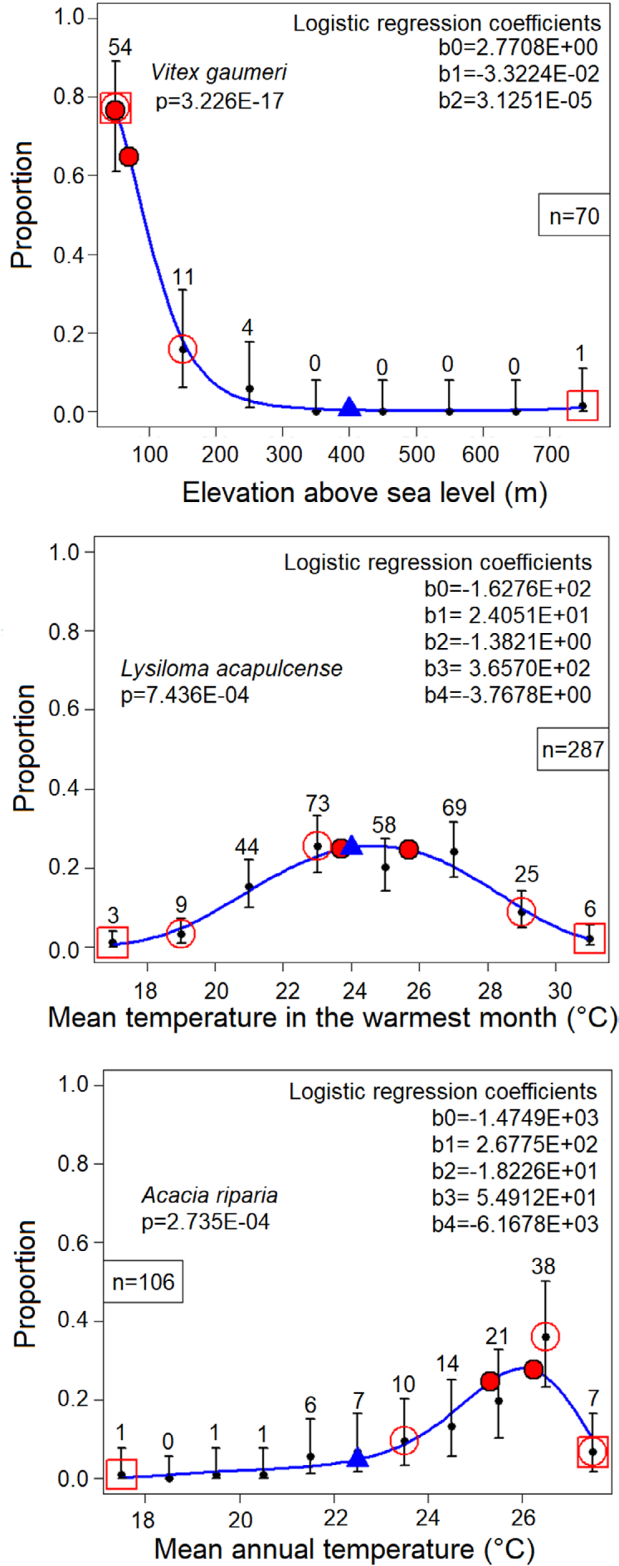
Three of 183 graphs that show different types of distribution shapes, resulting from the logistic regressions (blue line and regression coefficients $b_{i})$. Proportions were calculated from the detected tree frequencies, and can be interpreted as probabilities to find the tree species at that point of the environmental gradient. Vertical bars show 95% confidence intervals for the underlying proportions. The empty circles show significantly different points, and the empty squares the endpoints. The blue triangle is the midpoint of the environmental gradient, and red circles indicate the limits of the highest‐probability interval. The total number of data points (“*n*”) and the probability (“*p*”) of a chi‐squared test for differences among the proportions are also given.

The “global” chi‐square test was statistically significant for differences among mean proportions of a given graph in 80% of the species‐variable combinations (146 of 183). The corresponding probabilities of the 183 chi‐square tests (that the parametric proportions are the same in each case) ranged from $2.0\cdot 10^{-206}$ to 0.97, with a median of $5.6\cdot 10^{-5}$. In 37 cases, there were one or a few pairwise significant differences along the gradient, but they were not strong enough to make the chi‐squared test significant. In those cases, the overall evidence for significant differences of the proportions along the gradients is weaker.

### 3.1 Correlation Among the Environmental Variables

The absolute values of the 120 Pearson correlation coefficients for the 16 environmental variables ranged from 0.0005 to 0.915, with the median being 0.230 and the mean 0.278. For 100 pairwise comparisons (83%), the absolute values of the Pearson correlation coefficients were in the lower half $\left(r<0.5\right)$, and only in 20 cases (17%) were they in the upper half $\left(r\geq 0.5\right)$. In conclusion, very high correlations are rare in our analysis, and thus the grouping of variables that behave similarly in terms of distance to the midpoint, seems not to be a major issue.

### 3.2 Relative Distance of the Highest‐Probability Interval From the Variables' Midpoint

**FIGURE 4:**
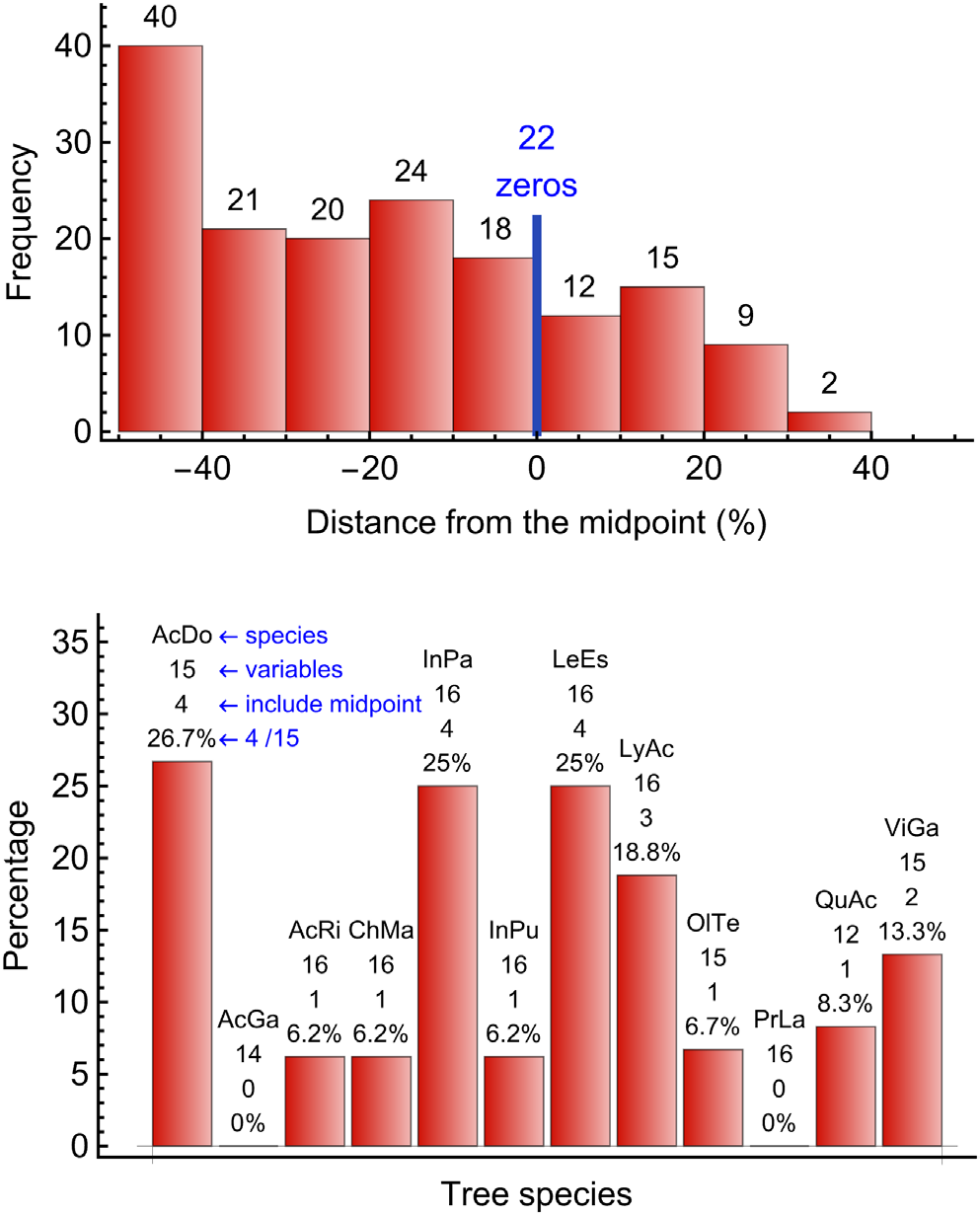
*Above*: Histogram of the relative distance of the highest‐probability interval from the midpoint for 161 species‐variable combinations. The highest‐probability intervals can be anywhere along the environmental gradient, with the maximum frequency found on the lower extreme $\left(-50\%\right)$. Only for 12% of the 183 species‐variable combinations, the midpoint was inside the highest‐probability interval, and thus the distance was 0% (22 cases, shown as a blue vertical line). *Below*: Percentage of variables for each species, for which the highest‐probability interval includes the midpoint. Above each bar, the species is indicated with the first two letters of the genus name together with the first two letters of the species epithet. Below the species abbreviation is the total number of environmental variables, followed by the number of variables, where the highest‐probability interval includes the midpoint, and the corresponding percentage.

Calculating the relative distance $\left(\text{relDis}\right)$ for all 183 species‐variable combinations, the midpoint falls into the highest‐probability interval (the relative distance is 0%) only in 12.0% of the cases (22 of 183). The distances are provided in the Supporting Information S1c. Figure 4 presents above a histogram of the relative distances of the highest‐probability interval from the midpoint for the 161 species‐variable combinations with a distance that was not 0%. In addition, the 22 cases at 0% are shown as a blue vertical line. The 161 cases involve all 12 species and all 16 variables, and the 22 cases include 10 different species and also 10 different variables. Consequently, the preference away from the midpoint is not a particular case of a few species or a few variables only. Furthermore, the highest‐probability intervals can be anywhere along the environmental gradient, with the maximum found even at the lower extreme $\left(-50\%\right)$. Considerably more species‐variable combinations had their highest‐probability intervals below the midpoint (123 cases) than above the midpoint (38 cases) on the environmental gradient. The difference is statistically highly significant in a binomial test, compared to an expectation of half of the cases on either side of the midpoint. We have not come up with an explanation for this observation. The already mentioned correlations among variables could possibly play a role, in that the grouping is such that the chosen variables are in the negative range.

Next, we calculated for each of the 12 species the number of variables for which the highest‐probability interval included the midpoints. This number can then be converted into a percentage of the total number of variables considered for the species. The 12 calculated proportions in Figure 4 below range from 0% (for two species) to 26.7%, with an average of 11.9% and a median of 7.5%. An average expectation of 11.9% per species for the highest‐probability intervals of all variables to include the midpoint indicates that this was the exception, rather than the rule.

### 3.3 Truncation of the Environmental Gradients

The detection of a species having the highest probability of occurrence around the midpoint of its environmental range requires that the whole range has been analyzed. Correspondingly, we calculated for each of the 161 nonsymmetrical species‐variable combinations, how much the shorter distance from the mode to the empirical limit of the range would have to be increased, in order to achieve symmetry around the highest‐probability interval. The result is shown in Table 2. It turned out that there were 100 species‐variable combinations (55% of 183) for which expansion to achieve symmetry is impossible. Only for 61 species‐variable combinations (33% of 183) could one search for data to expand the ranges' corresponding limit (those with “Yes” in the penultimate column of Supporting Information S1c), whereas 22 (12%) were centered to begin with. The impossibility of expanding ranges results from the truncation of the environmental gradients, where the ranges would have to be expanded. The truncation is a consequence of the nature of the variable. Of the 16 variables, 11 cannot be negative (such as precipitation), one variable cannot exceed 1, and only four variables are not numerically limited. The details of our analysis for each species‐variable combination are provided in Supporting Information S1c.

**TABLE 2:** Number of species for which the environmental gradient is truncated, making it impossible to increase the shorter distance from the mode to get symmetry around the peak of highest frequency (mode).

Variable	Midpoint in highest‐probability interval	Expansion possible	Expansion not possible	Comment in case of being truncated
Annual dryness index	1	2	9	Cannot be negative
Average slope of the terrain (°)	1	0	11	Cannot be negative
Cation exchange capacity of soil (cmolc/kg)	4	5	2	Cannot be negative
Elevation above sea level (m)	0	3	7	Cannot be negative
Mean annual precipitation (mm)	1	3	8	Cannot be negative
Mean annual temperature (°C)	4	8	0	—
Mean temperature in the coldest month (°C)	3	6	0	—
Mean temperature in the warmest month (°C)	2	9	0	—
Ratio of summer to total precipitation	2	3	7	Cannot be > 1
Soil organic‐carbon content (g/kg)	0	1	11	Cannot be negative
Soil's pH value	2	10	0	—
Spring precipitation (mm)	0	2	8	Cannot be negative
Summer precipitation (mm)	0	3	9	Cannot be negative
Summer precipitation balance	0	5	7	Cannot be negative
Summer/spring precipitation‐balance	0	0	12	Cannot be negative
Winter precipitation (mm)	2	1	9	Cannot be negative
Total	22	61	100	

### 3.4 Expansion Factor Required for Symmetry

**FIGURE 5:**
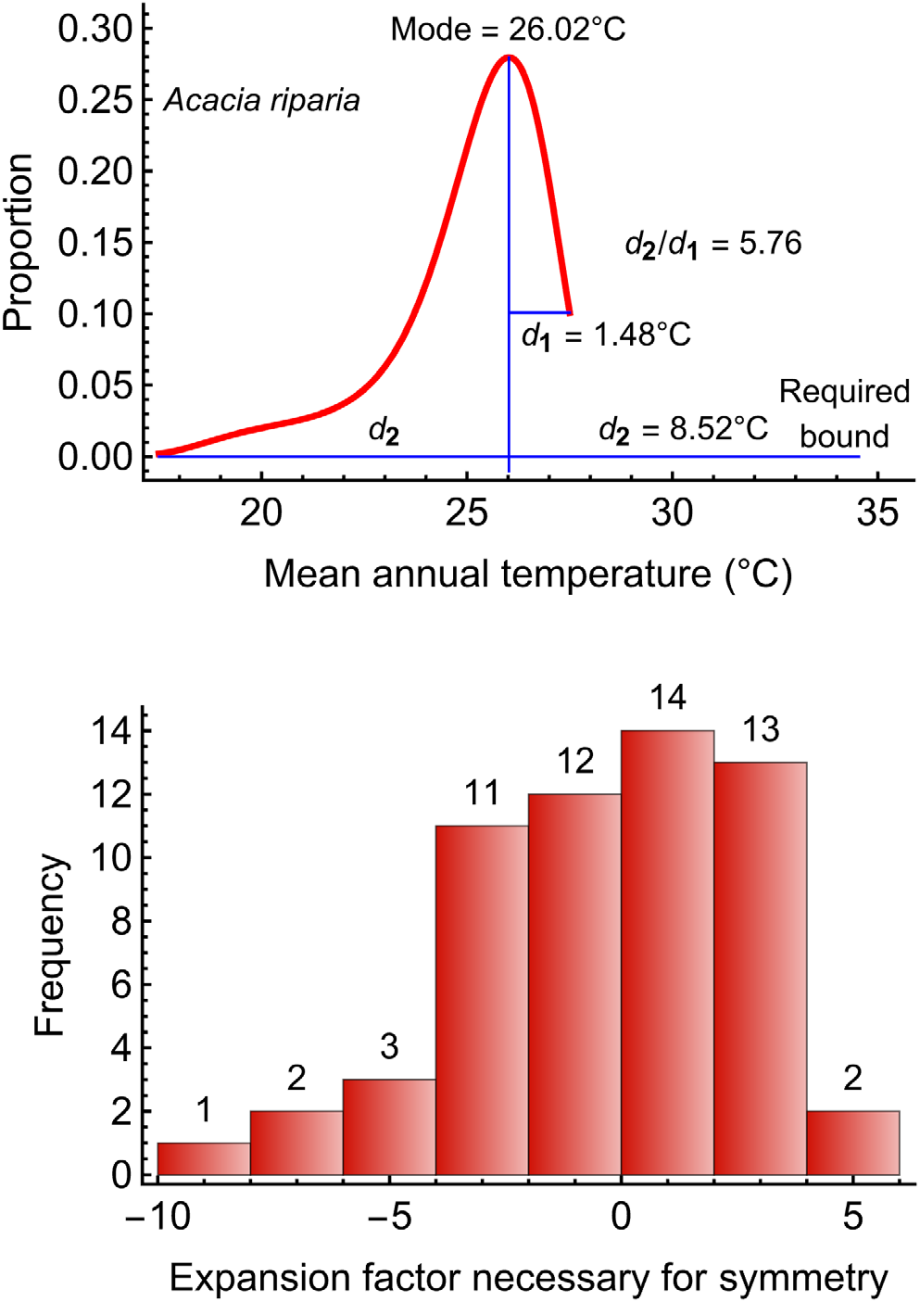
Relative distances of hypothetical limits for symmetry, for variables with statistical distributions that are not truncated. *Above*: The encountered lower limit of mean annual temperature for *Acacia riparia* was 17.5°C and the upper limit was 27.5°C. The mode was not found at the midpoint (22.5°C), but at 26.02°C. For lying symmetrically at the midpoint of the limits, the upper limit would need to be 34.54°C. This is 5.76 times larger than the encountered distance from the mode (1.48°C). *Below*: Expansion factors for 58 variables, whose statistical distributions are not symmetrical, but expansion is theoretically possible. The expansion factor is the distance that would be required for symmetry (*d*_2_) divided by the distance of the mode to the nearest extreme (*d*_1_), positive to the right and negative to the left. The range is from −8.00 to 5.76, with a median of 0.12.

In the previous section, the shorter tail of the environmental gradients could theoretically be expanded for 61 species‐variable combinations (33% of 183). Does the environmental center‐periphery hypothesis apply in those cases? Figure 5 shows above what we define as “expansion factor”: the distance that would be required for symmetry (*d*_2_) divided by the distance of the mode to the nearest extreme (*d*_1_), positive to the right and negative to the left. For example, the encountered lower limit of mean annual temperature for *Acacia riparia* was 17.5°C, and the upper limit was 27.5°C. The mode was not found at the midpoint (22.5°C), but at 26.02°C. For lying symmetrically at the midpoint of the limits, the upper limit would need to be 34.54°C. This is 5.76 times larger than the encountered distance from the mode (1.48°C). Supporting Information S1c (last column) provides the calculation of each expansion factor.

The 58 expansion factors include 31 cases without truncated environmental gradients, but also 27 cases with truncated ones without reaching the truncated extreme, and three cases, for which the expansion factors could not be calculated. In the latter cases, the encountered mode is at the lower or upper extreme, so that $d_{1}=0$, and the true mode is unknown. Thus, truncation does not imply necessarily that symmetry is impossible. However, given that truncation makes symmetry impossible for over half (100 of 183) of the species‐variable combinations, it is obviously a rather frequent situation.

A final issue is to look at the minimum or maximum parameters required for symmetry, in order to see if they are feasible in the cases, where they are theoretically possible. First, one can ask, if the parameters required for symmetry exist in Mexico. Second, one could investigate if the studied species could exist on the sites where those extreme parameters are found. Table 3 presents data to partially answer the first question. For 14 environmental variables, the farthest outlying required parameters encountered for any species (minimum and/or maximum) are shown (third column). The species, for which the extreme applies, is given in parentheses. In nine cases, the environmental gradients are truncated but not within the required range (second column). Finally, in the last column, the required parameters are compared with the overall ranges found for all species in Supporting Information S1b. According to that comparison, 5 of 12 minima (56%) and 4 of 9 maxima (44%) exist in Mexico. The remaining extremes could exist, but further research would be required to continue this analysis.

**TABLE 3:** Extreme parameters required for the environmental variables in our study.

Variable and species	Truncated variable?	Needed	Within range in S1b?
Expansion to the left of the mode :			
Annual dryness index (*Acacia gaumeri*)	Yes (≥ 0)	0.69	No
Cation exchange capacity of soil ( *Prosopis laevigata* )	Yes (≥ 0)	1.5 cmolc/kg	No
Mean annual precipitation (*Vitex gaumeri*)	Yes (≥ 0)	107 mm	Yes
Mean annual temperature ( *Prosopis laevigata* )	No	6.0°C	No
Mean temperature in the coldest month ( *Prosopis laevigata* )	No	−0.7°C	No
Mean temperature in the warmest month ( *Prosopis laevigata* )	No	8.7°C	No
Ratio of summer to total precipitation (*Inga pavoniana*)	Yes (≥ 0, ≤ 1)	0.43	Yes
Soil organic‐carbon content ( *Leucaena esculenta* )	Yes (≥ 0)	1.5 g/kg	Yes
Soil's pH value (*Inga punctata*)	No	3.9	No
Summer precipitation (*Acacia gaumeri*)	Yes (≥ 0)	57 mm	Yes
Summer precipitation balance (*Inga punctata*)	Yes (≥ 0)	0.25	No
Winter precipitation (*Acacia gaumeri*)	Yes (≥ 0)	28 mm	Yes
**Expansion to the right of the mode:**			
Cation exchange capacity of soil (*Lysiloma acapulcense*)	Yes (≥ 0)	40 cmolc/kg	Yes
Elevation above sea level ( *Prosopis laevigata* )	Yes (≥ 0)	3491 m	No
Mean annual precipitation (*Acacia dolichostachya*)	Yes (≥ 0)	1699 mm	Yes
Mean annual temperature ( *Acacia riparia* )	No	34.5°C	No
Mean temperature in the coldest month (*Chloroleucon mangense*)	No	34.7°C	No
Mean temperature in the warmest month ( *Acacia riparia* )	No	35.8°C	No
Ratio of summer to total precipitation (*Acacia gaumeri*)	Yes (≥ 0, ≤ 1)	0.83	Yes
Soil's pH value ( *Prosopis laevigata* )	No	9.1	No
Spring precipitation (*Acacia dolichostachya*)	Yes (≥ 0)	225 mm	Yes

## 4 Discussion

The literature about the environmental center‐periphery hypothesis has focused largely on evidence from empirical analyses; Dallas et al. (2020: 82) concluded that around 20% of the studied species would support the hypothesis. Santini et al. (2019: 696) stated that the hypothesis “so far remains an appealing speculation with little and variable empirical support.” As in our study of tree species, a typical result for different species is mixed support, such as by Waldock et al. (2019: 685) for the abundance of 702 reef fish species and water temperature. The discussions about the reasons address many topics, from measurement issues (Sagarin and Gaines 2002; Borregaard and Rahbek 2010; Yañez‐Arenas et al. 2014; Pironon et al. 2017; Yañez‐Arenas et al. 2020), dispersal limitations (Osorio‐Olvera et al. 2019; Dallas et al. 2020), and population genetics (Lira‐Noriega and Manthey 2014; Pironon et al. 2017; Cruz‐Nicolás et al. 2020), to climate change and the ongoing adaptation to the environment (Toledo et al. 2012; Dallas et al. 2020). In a theoretical simulation study with a defined mathematical model, Osorio‐Olvera et al. (2019: 1419) concluded that “dispersal, barriers, transitory states, and Allee effects” are important. Yañez‐Arenas et al. (2014: 37) addressed sampling issues, based on a multivariate normal distribution for abundances of a virtual species, which assumes symmetry around a centroid, and normality of the statistical distribution to start with. The observation and distinction of natural truncation of environmental gradients have rarely been made. Austin and Nicholls (1997) touch on the issue of truncation, but do not discuss its wider implications for the environmental center‐periphery hypothesis. Martin and Canham (2020) seem to encounter also the issue of truncation for precipitation, without explicitly stating or discussing it (their Figure 3B).

Our data of 183 species‐variable combinations has many of the same limitations that are already treated in the literature, such as potentially incomplete range representation for the whole environmental gradient, but reveals an important conceptual aspect that apparently has never been discussed. There were 100 species‐variable combinations (55% of 183), for which symmetrical peaks are impossible, 61 (33%) for which one could search for data to expand one of two ranges' limits, and only 22 (12%) with approximate symmetry (the midpoint falls into the highest‐probability interval). Our conclusion is not that the environmental center‐periphery hypothesis is refuted, but that it has to be conditioned, such that natural truncation of the environmental gradient is not limiting on one side (or both sides) of the mode.

The theoretical basis for the environmental center‐periphery hypothesis is that a species would develop its highest frequency of occurrences at a combination of environmental parameters in a niche, to which it is best adapted. Moving away from this “best combination” could cause a decrease, for a number of reasons: less available nutrition, more enemies, more diseases, less adequate habitat for reproductions, *et cetera*. At some distance from the best combination, a species is not able to survive anymore (Maguire 1973: 215–216). Sagarin & Gaines (2002: 137) state the idea in the following way: “A widely held belief in biogeographical ecology is that a species' abundance is typically greatest at the centre of its geographical range and uniformly low toward the edges.” Thus, the “expansion possibility” is expected to decrease symmetrically on both sides of the highest‐probability point on the environmental gradient. Subsequently, and also here, not a geographic but an environmental gradient has been considered. Accordingly, several analyses are based on the assumption of a symmetrical (bell‐shaped) normal (Gaussian) distribution (Canham and Thomas 2010: 3434, Yañez‐Arenas et al. 2014: 37, Osorio‐Olvera et al. 2016: 1082). A minor point is that statistical kurtosis and alternative symmetrical distributions are rarely discussed. More importantly, symmetry itself is questionable (Austin 1987: 35). Why should it be expected that (for example) a 5°C higher mean temperature causes the same decrease in frequency like a 5°C lower mean temperature? This was already recognized by Maguire (1973: his Figure 1). Consequently, asymmetrical statistical distributions over an environmental gradient are naturally likely. Furthermore, one has to think of the mathematical domain of an environmental variable, such as nonnegativity. In a situation, where the environmental gradient simply stops for the species on one side (or both), the “expansion possibility” does stop as well. That, however, does not necessarily imply that the species “moves away” from the truncated limit. Consider a land species that has evolved near sea level, where it may present maximum fitness. Obviously, it cannot expand below sea level, but it can expand into higher altitudes (towards and beyond the midpoint), where the species would be expected to decrease its frequency. The result would *not* be an environmental gradient with maximum fitness and frequency at its midpoint. In such cases of truncated environmental gradients, the highest‐probability point could only by an unlikely coincidence fall exactly halfway in between the limits. More likely is that on one side of the environmental gradient, the expansion possibility is cut short, and on the other side it is freely available. An asymmetrical distribution over the gradient may result. In conclusion, for future studies it makes sense to use a conditioned environmental center‐periphery hypothesis that we state in the following way: “On an environmental gradient, a given species is able to cover a certain range. For environmental gradients, where natural truncation of the environmental gradient is not limiting for a given species, the highest probability of occurrences is found away from the range's endpoints, tending towards its midpoint.” This result has obvious implications for biogeographical assumptions, such as where to find the best areas for conservation or how to predict the effects of climate change.

## Author Contributions

**Pablo Antúnez:** data curation (lead), formal analysis (equal), investigation (equal), methodology (supporting), software (equal), validation (equal), visualization (lead), writing – original draft (supporting), writing – review and editing (supporting). **Martin Ricker:** conceptualization (lead), data curation (supporting), formal analysis (equal), investigation (equal), methodology (lead), software (equal), validation (equal), visualization (supporting), writing – original draft (lead), writing – review and editing (lead).

## Conflicts of Interest

The authors declare no conflicts of interest.

## Supporting information


Data S1.

## Figures and Tables

**FIGURE D1:**
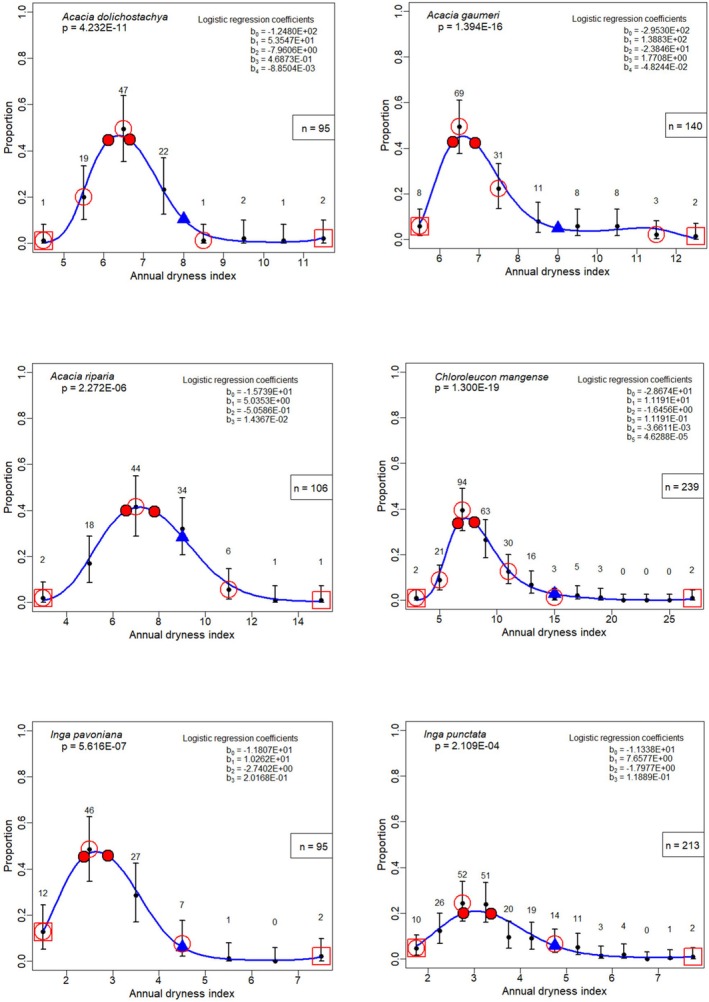
Occurrences along an environmental gradient of **annual dryness index**.

**FIGURE D2:**
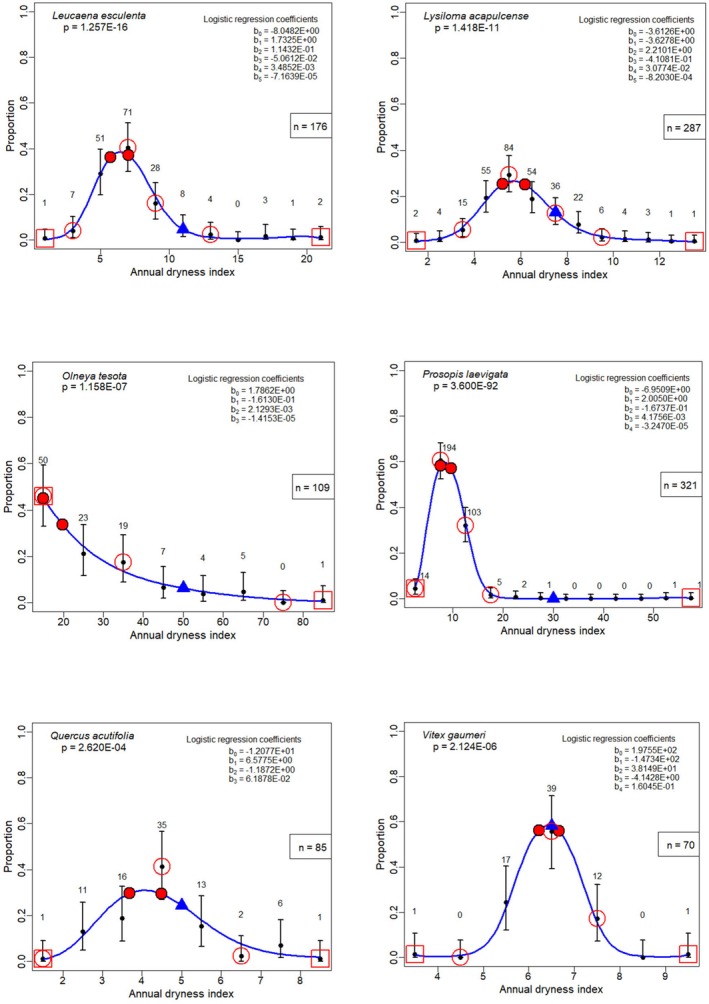
Occurrences along an environmental gradient of **annual dryness index** (continued).

**FIGURE D3:**
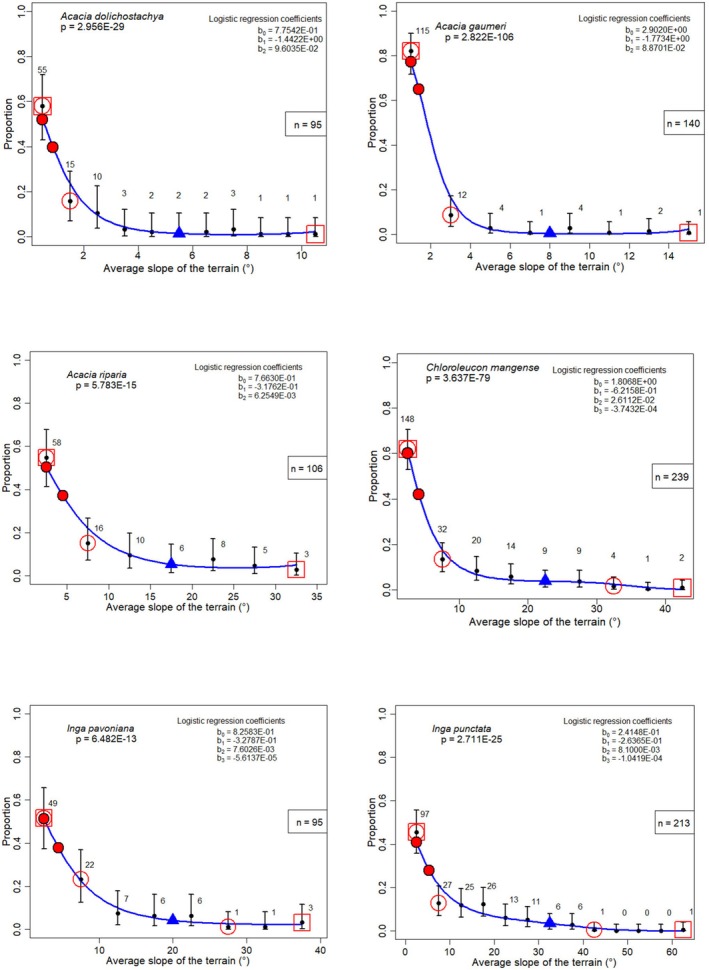
Occurrences along an environmental gradient of **average slope of the terrain**.

**FIGURE D4:**
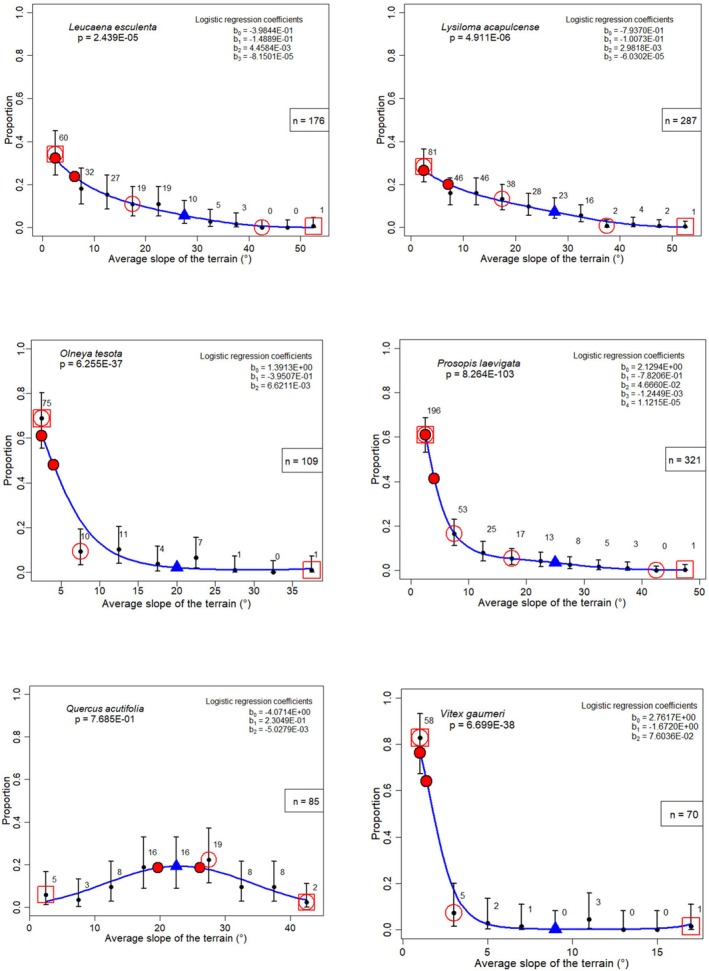
Occurrences along an environmental gradient of **average slope of the terrain** (continued).

**FIGURE D5:**
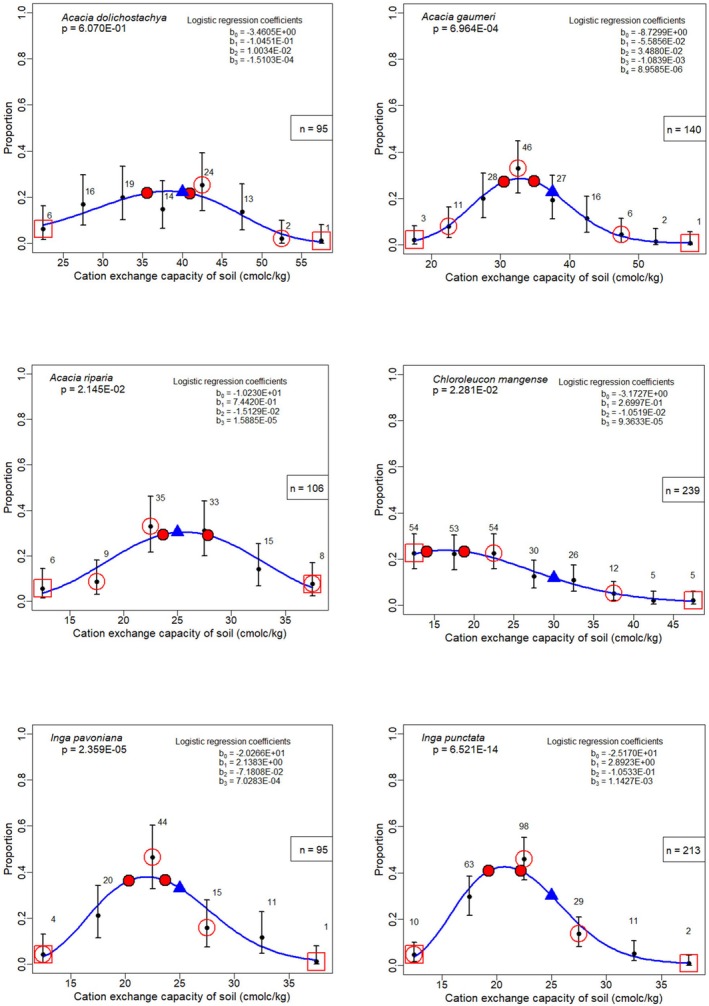
Occurrences along an environmental gradient of **cation exchange capacity of soil**.

**FIGURE D6:**
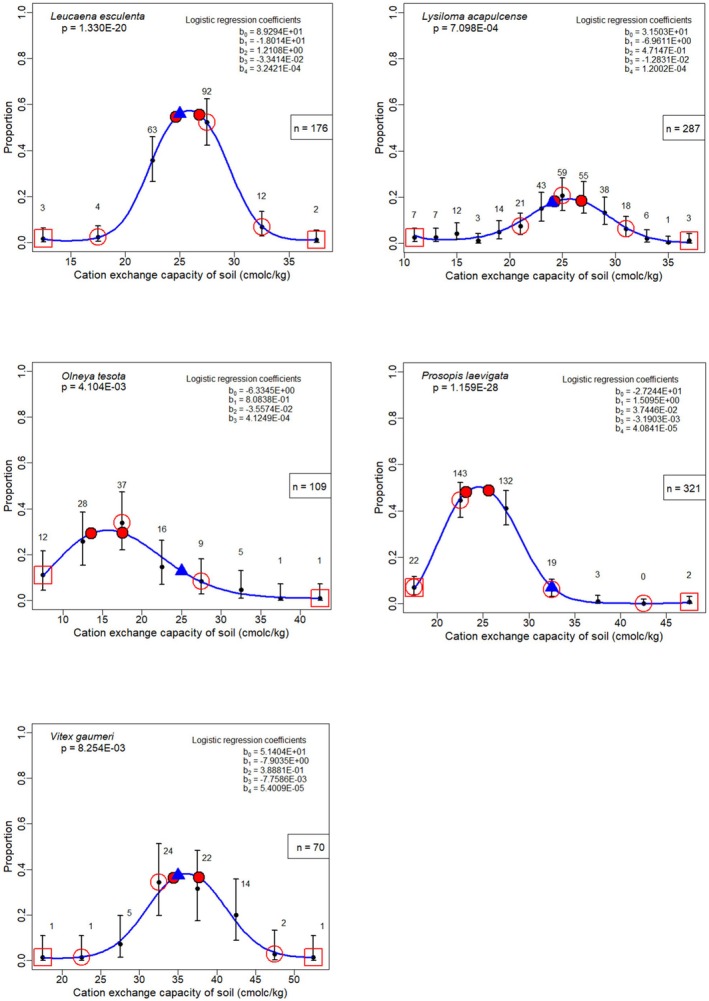
Occurrences along an environmental gradient of **cation exchange capacity of soil** (continued).

**FIGURE D7:**
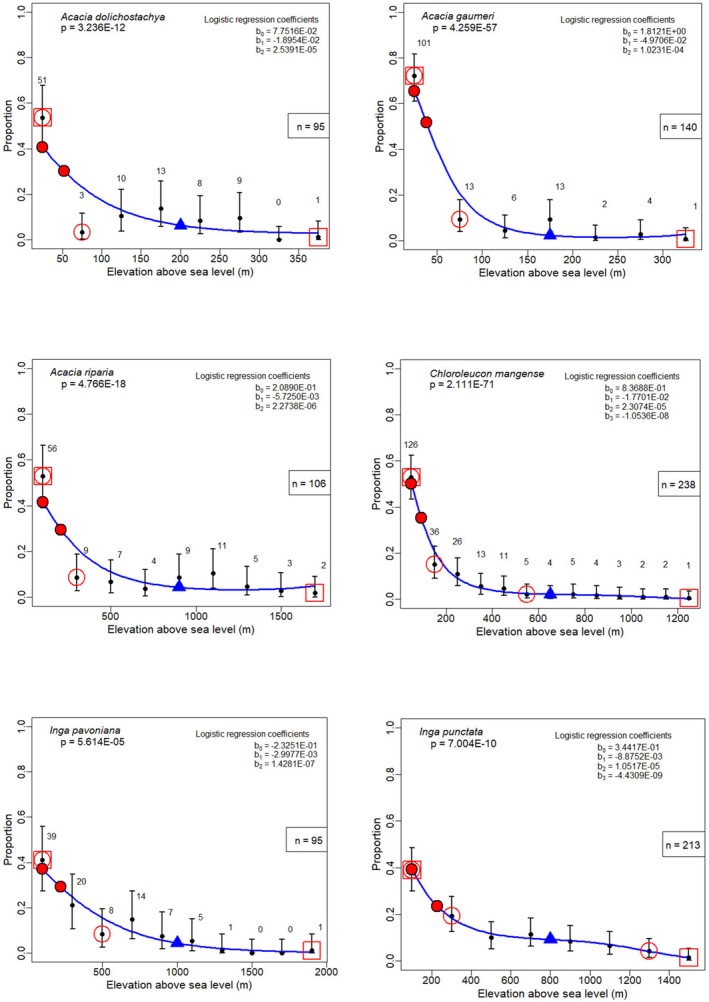
Occurrences along an environmental gradient of **elevation above sea level**.

**FIGURE D8:**
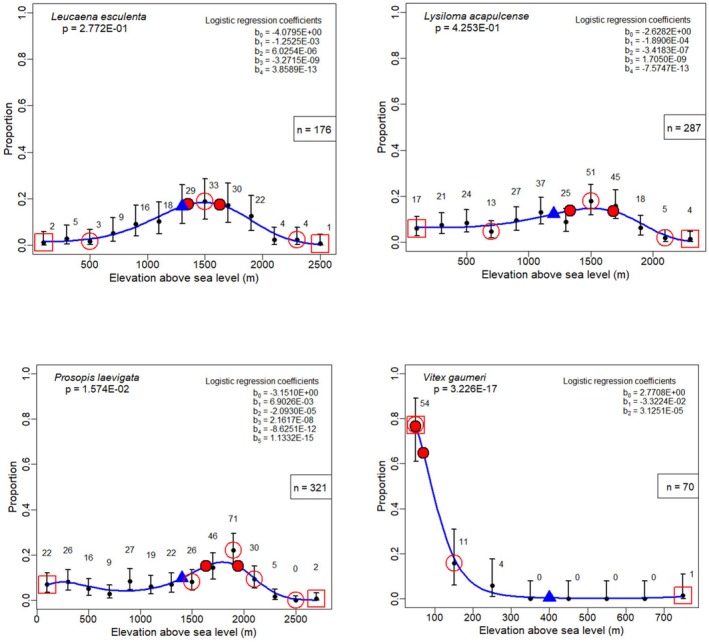
Occurrences along an environmental gradient of **elevation above sea level** (continued).

**FIGURE D9:**
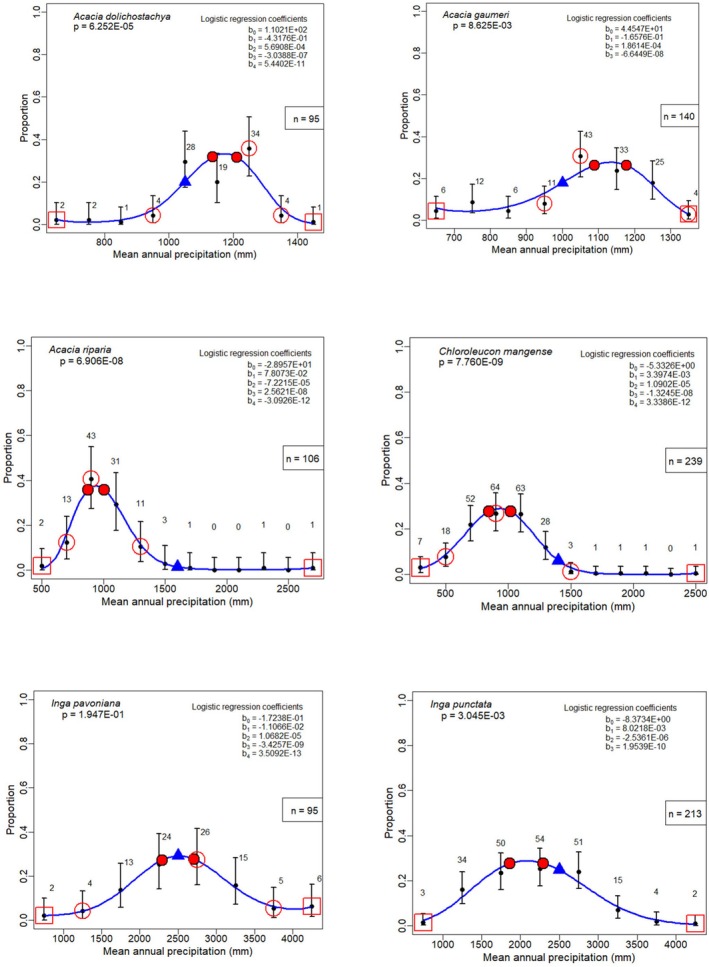
Occurrences along an environmental gradient of **mean annual precipitation**.

**FIGURE D10:**
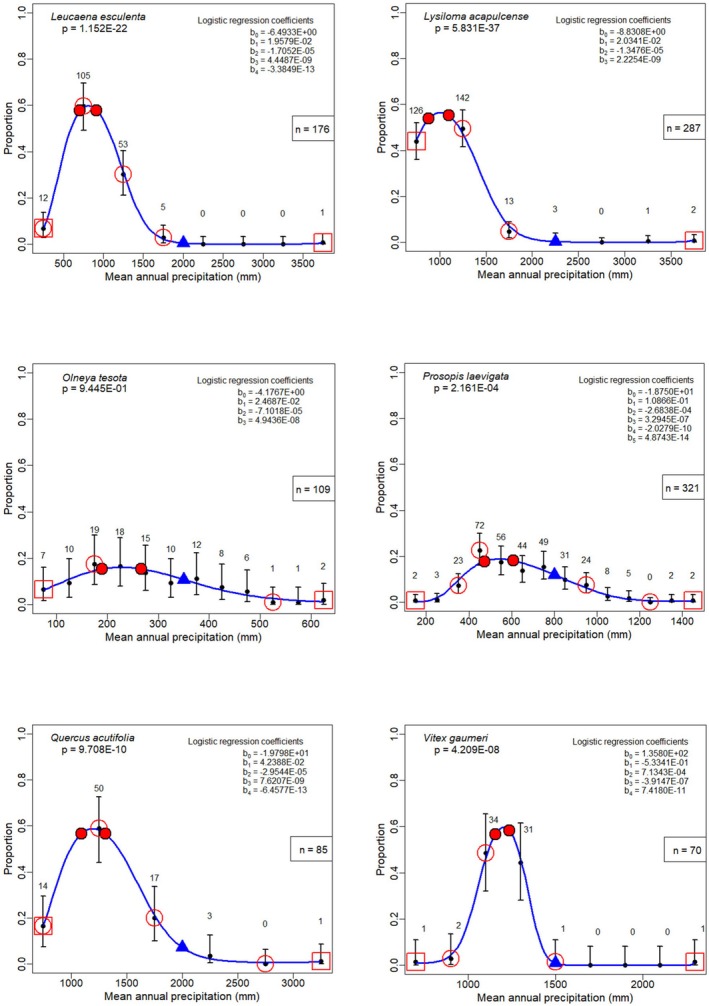
Occurrences along an environmental gradient of **mean annual precipitation** (continued).

**FIGURE D11:**
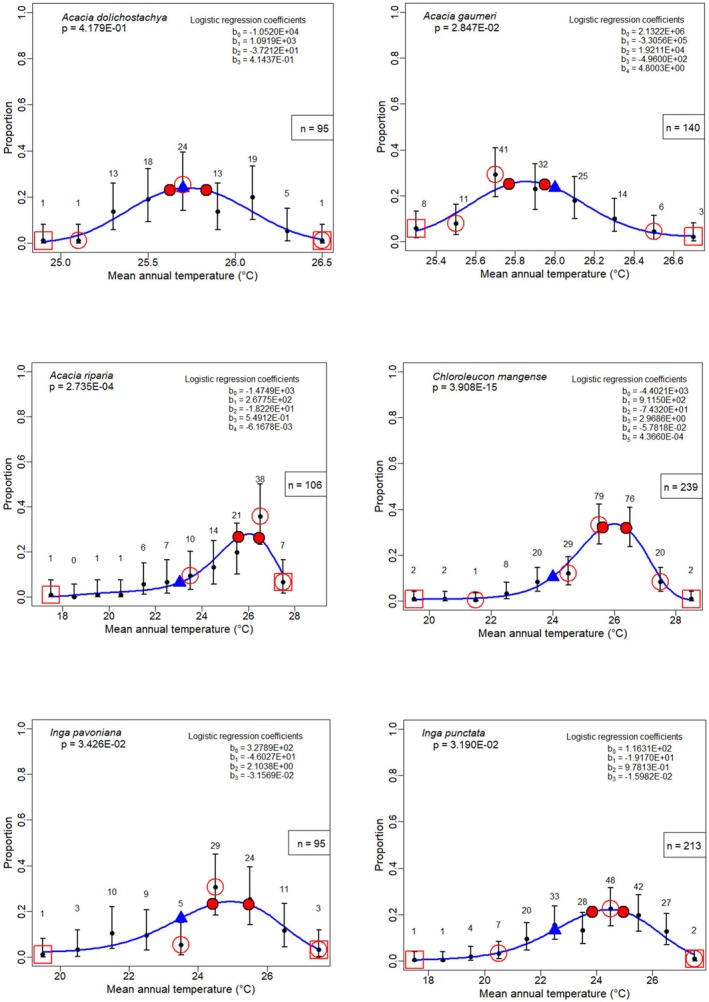
Occurrences along an environmental gradient of **mean annual temperature**.

**FIGURE D12:**
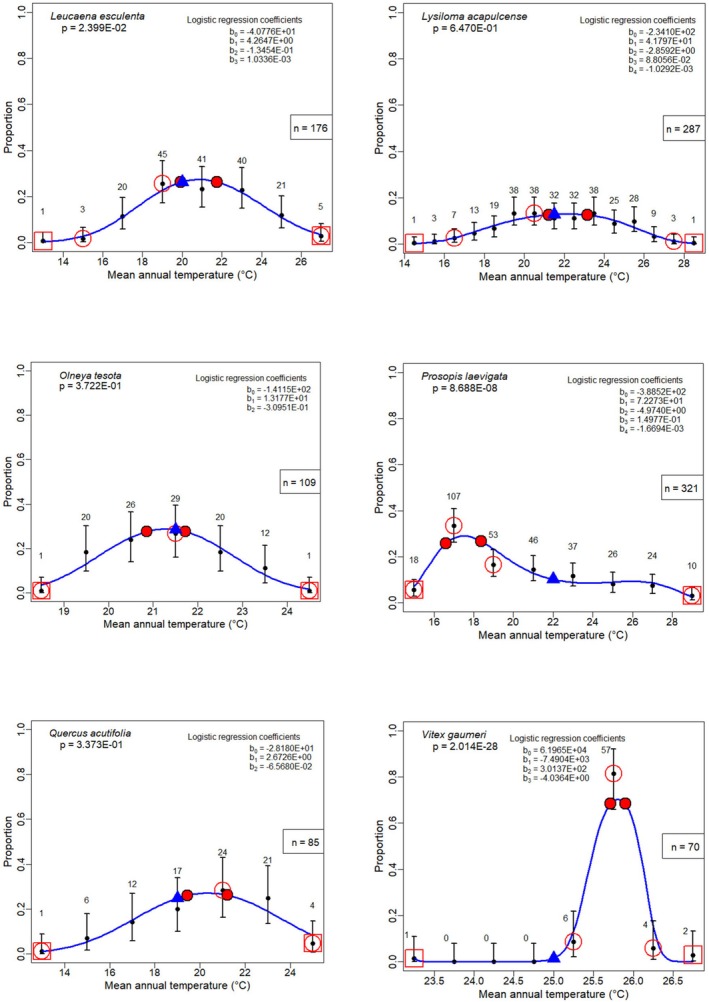
Occurrences along an environmental gradient of **mean annual temperature** (continued).

**FIGURE D13:**
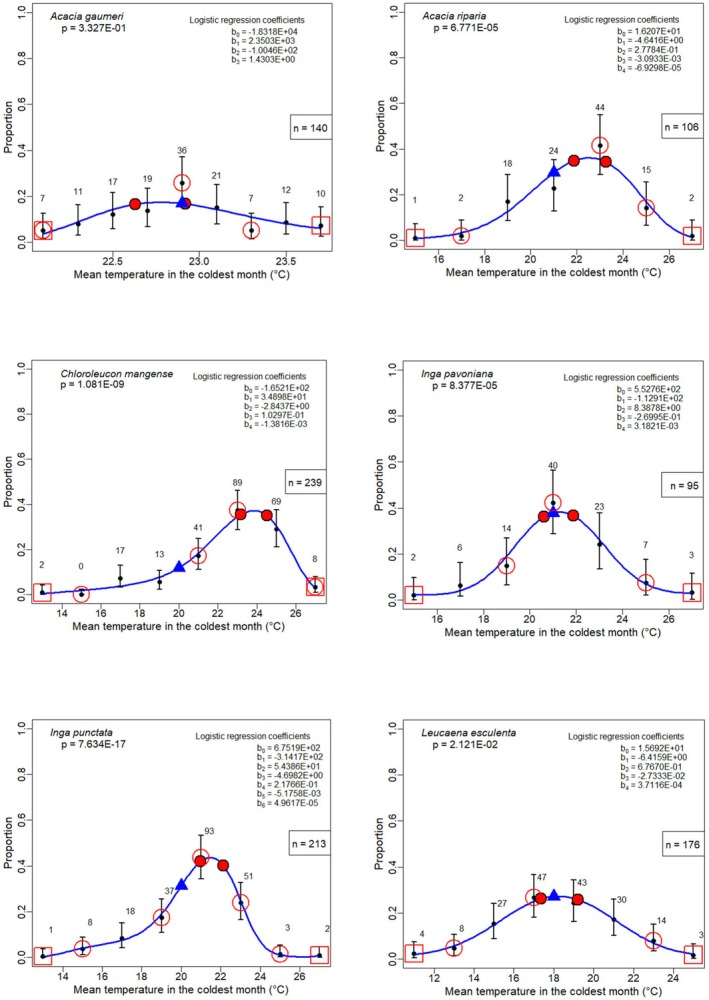
Occurrences along an environmental gradient of **mean temperature in the coldest month**.

**FIGURE D14:**
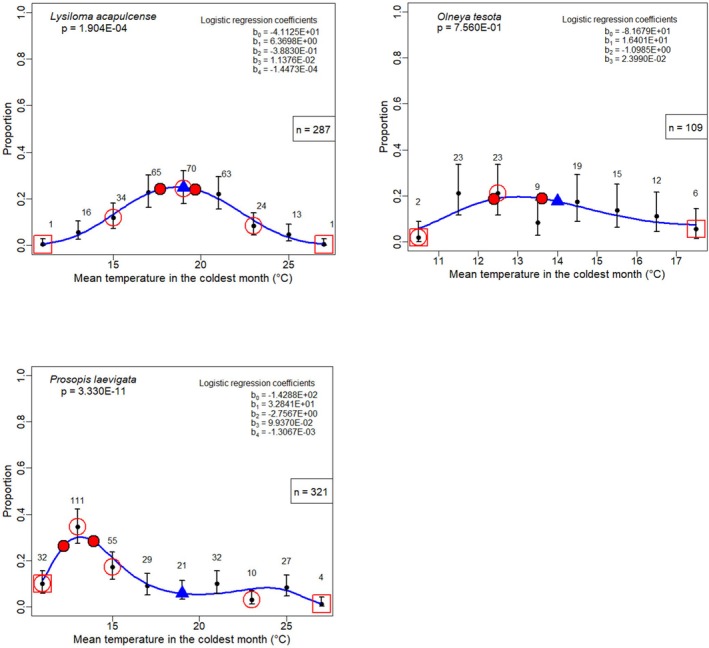
Occurrences along an environmental gradient of **mean temperature in the coldest month** (continued).

**FIGURE D15:**
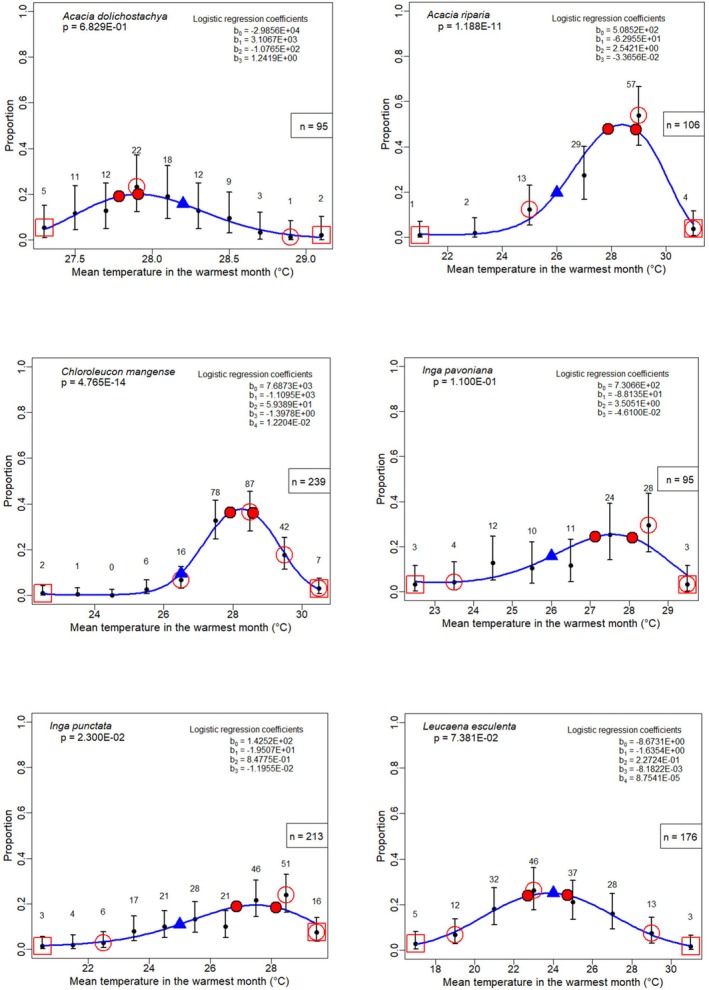
Occurrences along an environmental gradient of **mean temperature in the warmest month**.

**FIGURE D16:**
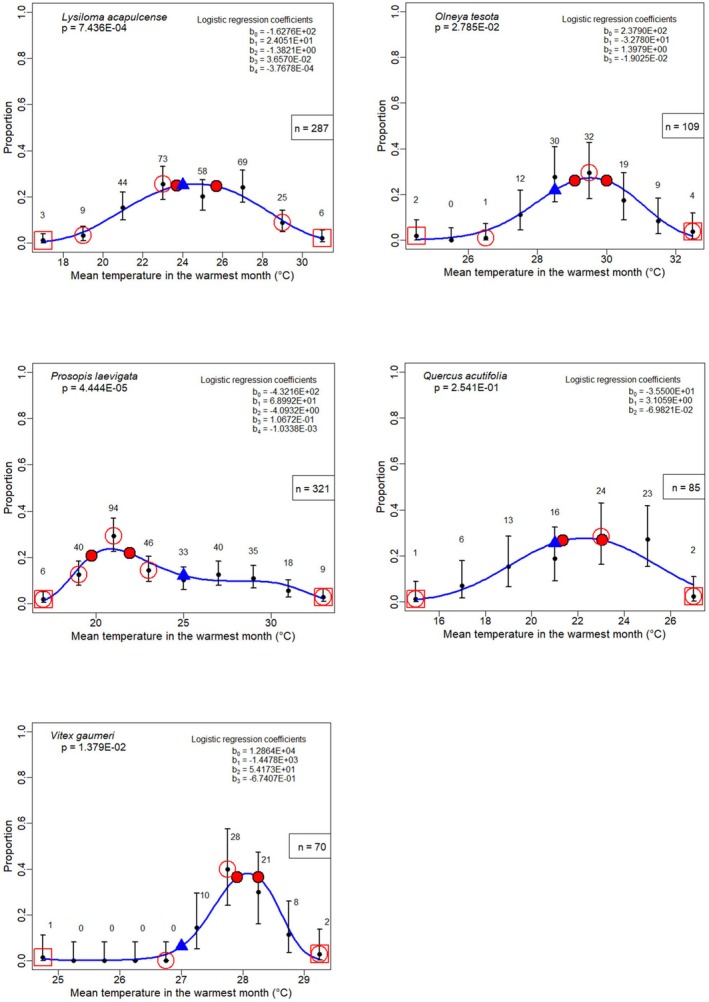
Occurrences along an environmental gradient of **mean temperature in the warmest month** (continued).

**FIGURE D17:**
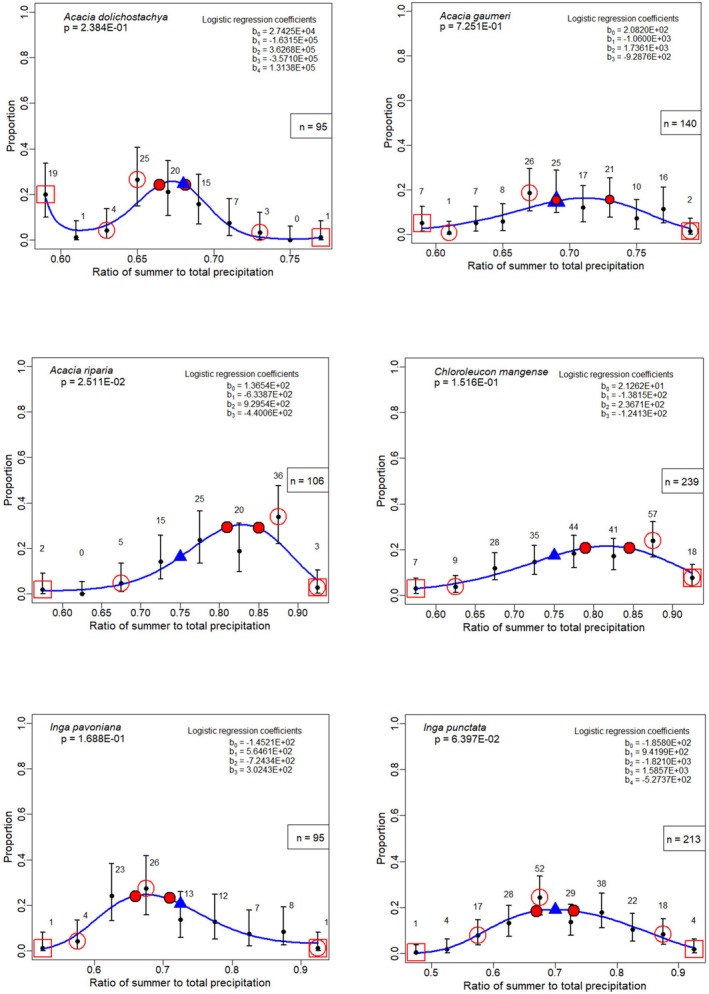
Occurrences along an environmental gradient of **ratio of summer to total precipitation**.

**FIGURE D18:**
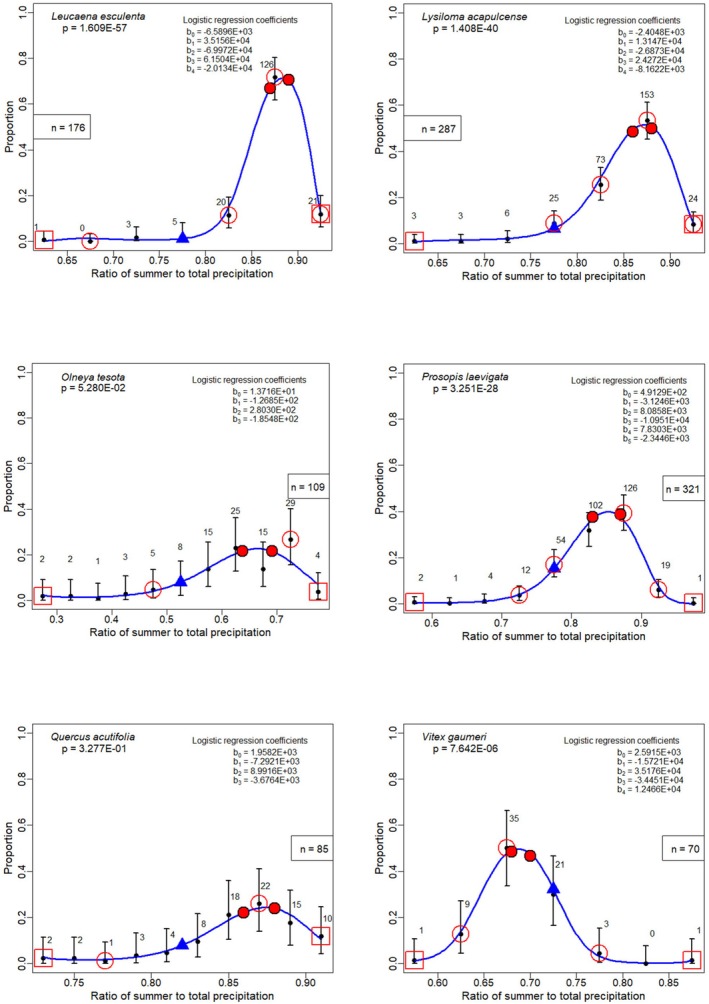
Occurrences along an environmental gradient of **ratio of summer to total precipitation** (continued).

**FIGURE D19:**
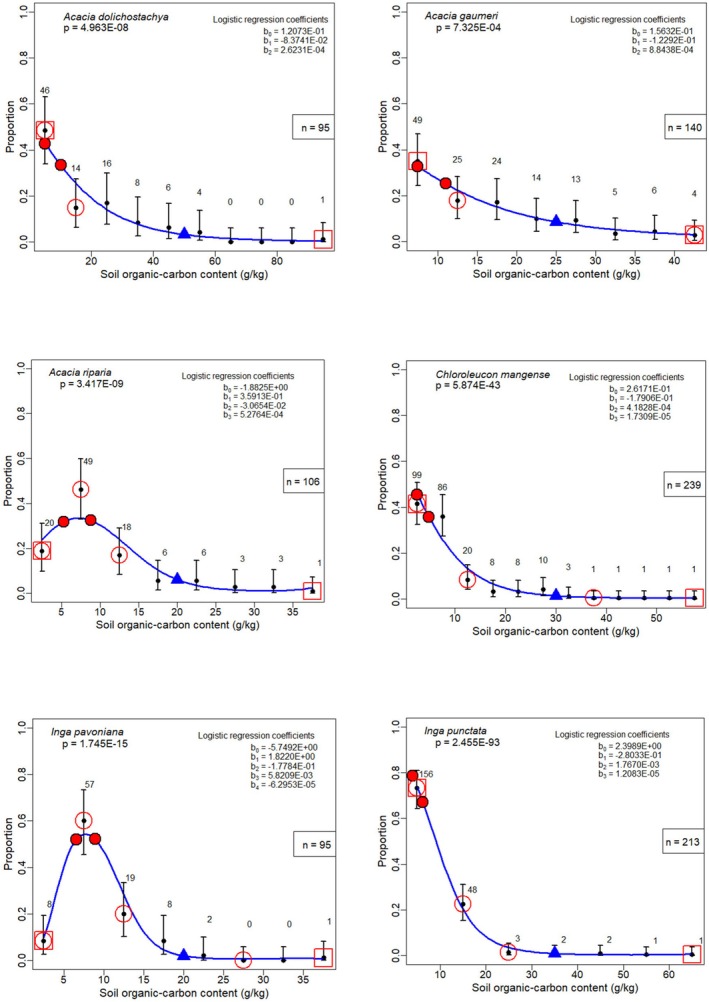
Occurrences along an environmental gradient of **soil organic‐carbon content**.

**FIGURE D20:**
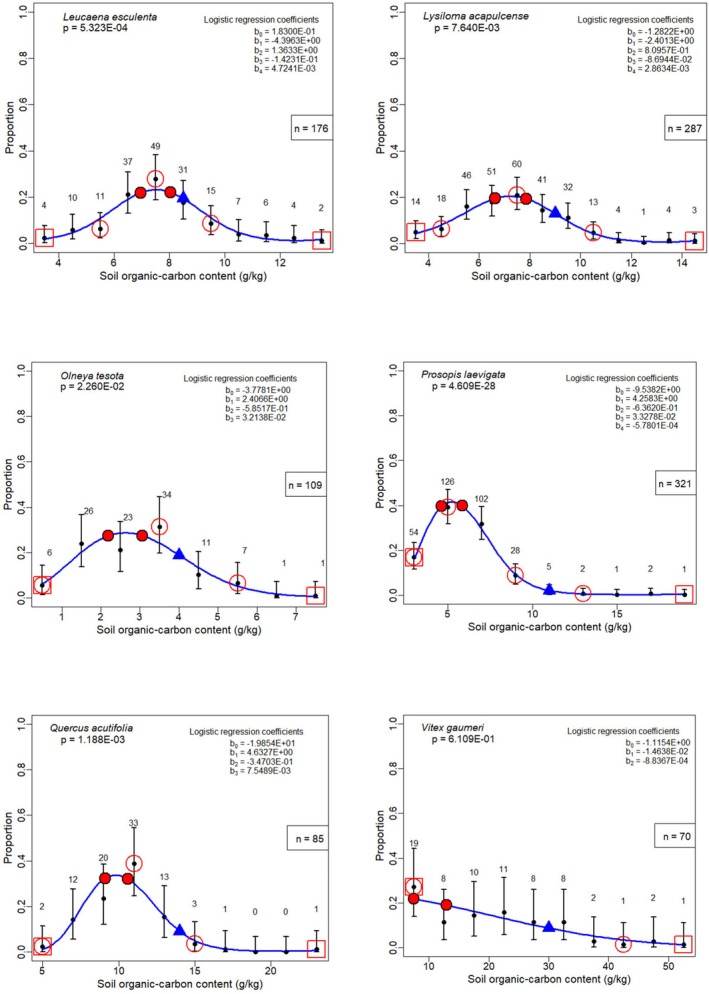
Occurrences along an environmental gradient of **soil organic‐carbon content** (continued).

**FIGURE D21:**
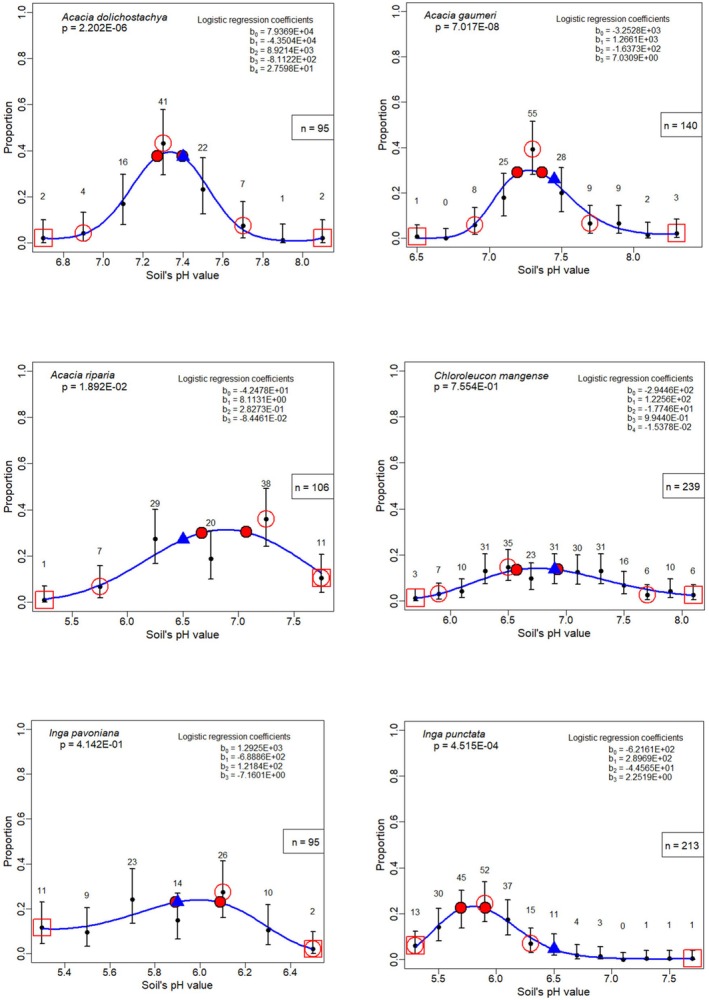
Occurrences along an environmental gradient of **soil's pH value**.

**FIGURE D22:**
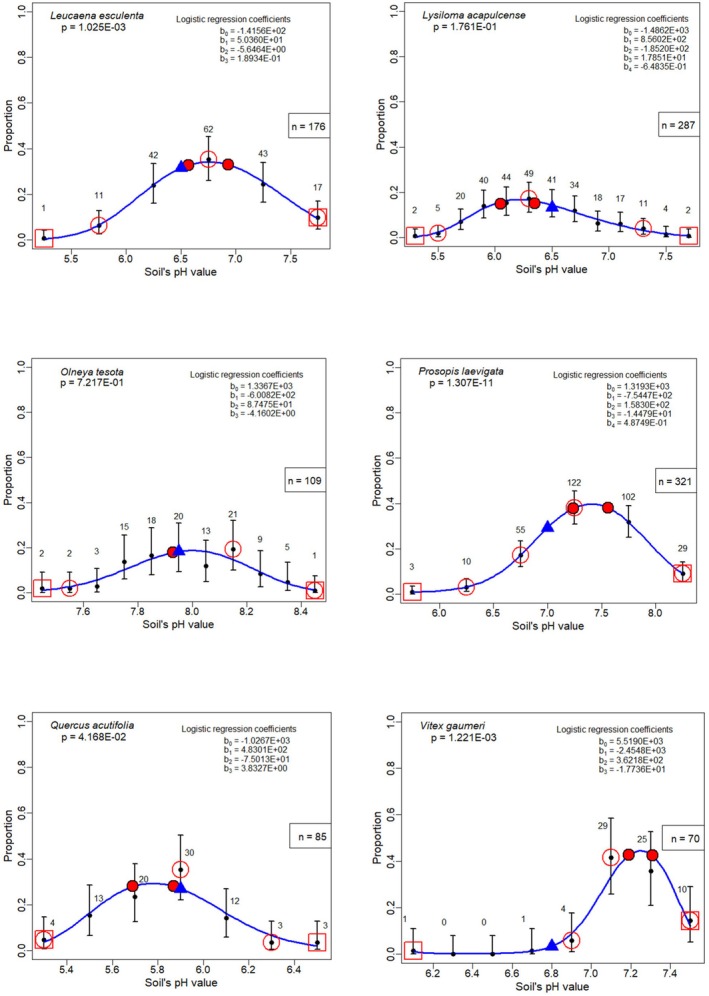
Occurrences along an environmental gradient of **soil's pH value** (continued).

**FIGURE D23:**
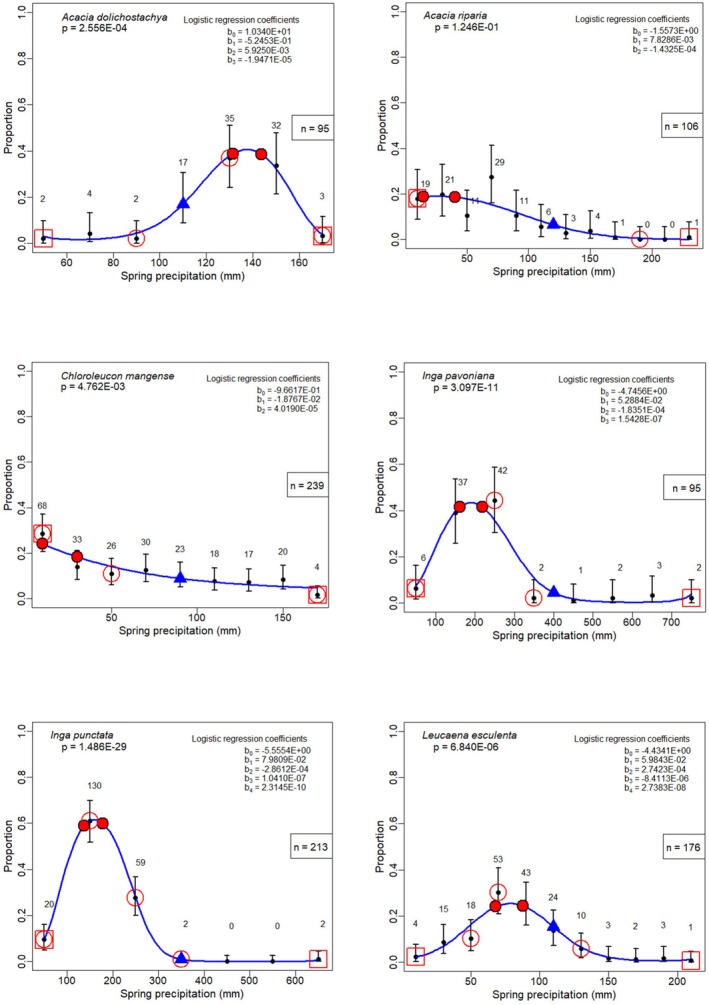
Occurrences along an environmental gradient of **spring precipitation**.

**FIGURE D24:**
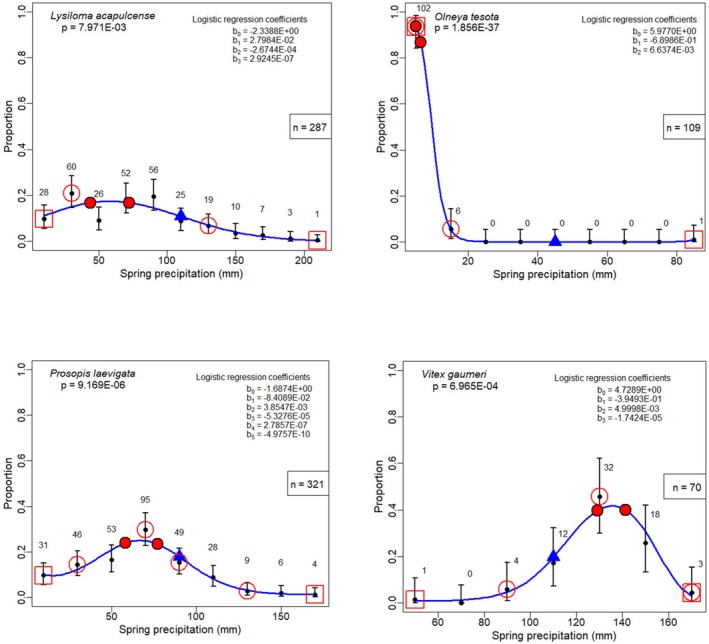
Occurrences along an environmental gradient of **spring precipitation** (continued).

**FIGURE D25:**
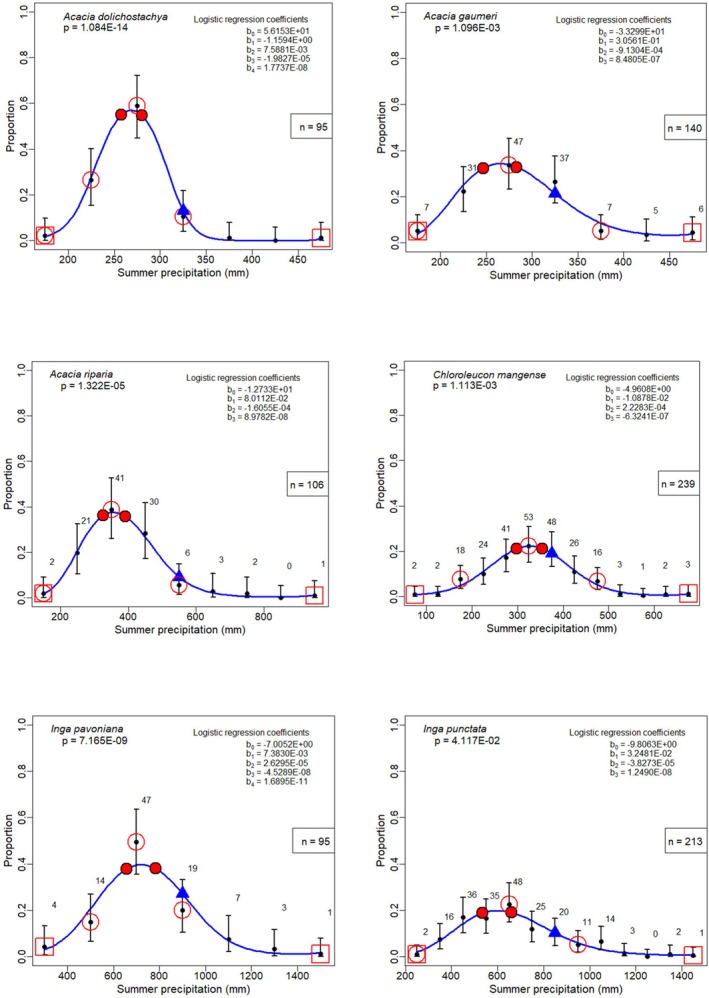
Occurrences along an environmental gradient of **summer precipitation**.

**FIGURE D26:**
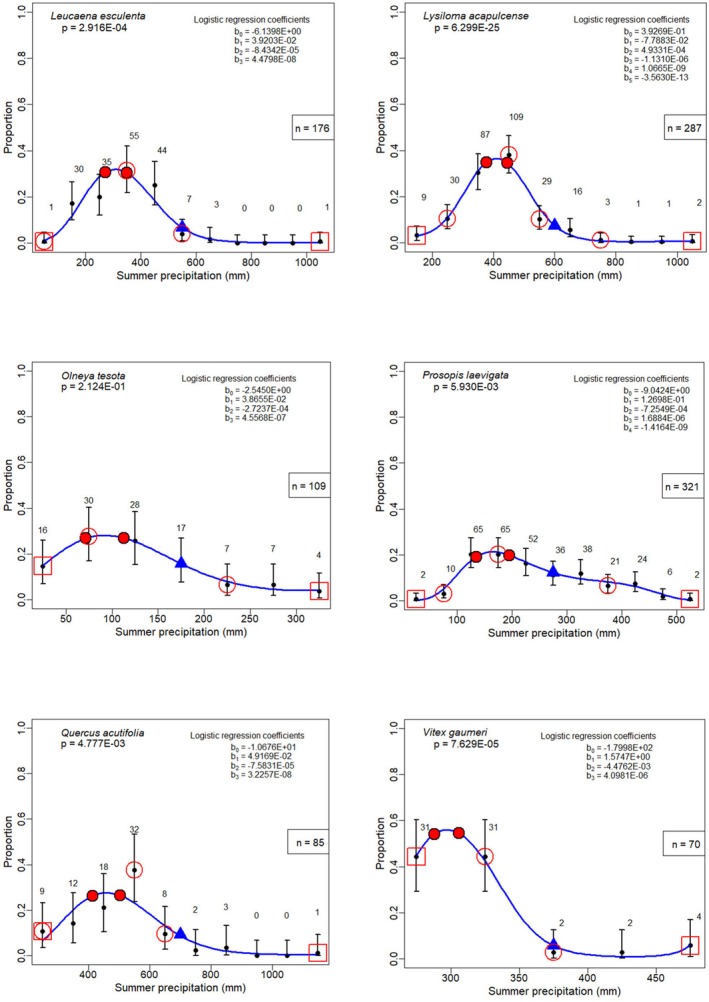
Occurrences along an environmental gradient of **summer precipitation** (continued).

**FIGURE D27:**
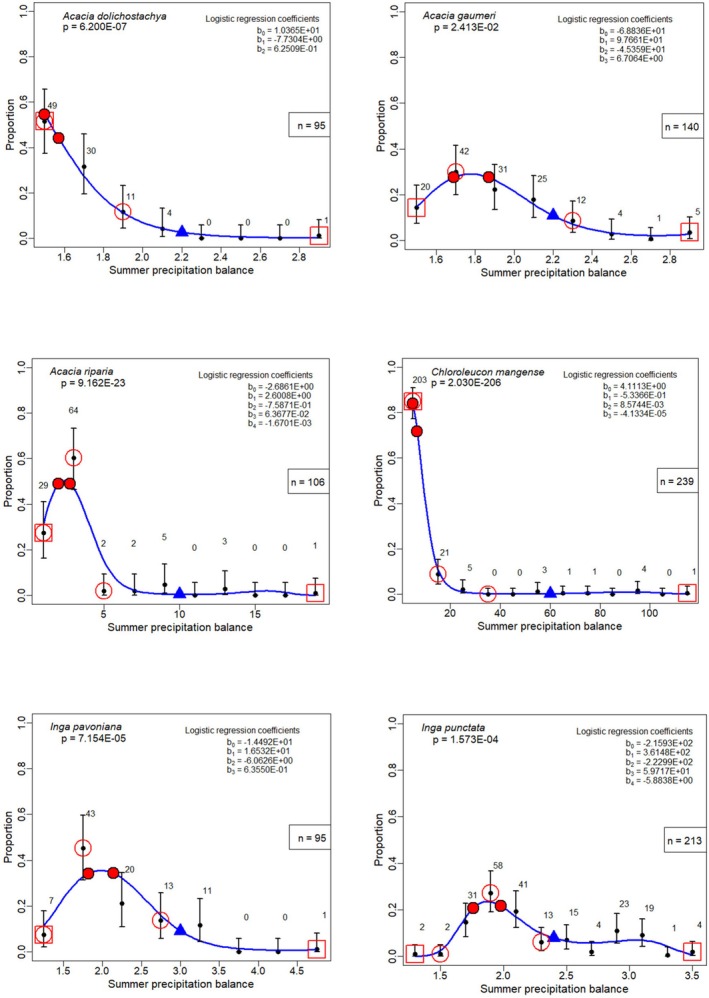
Occurrences along an environmental gradient of **summer precipitation balance**.

**FIGURE D28:**
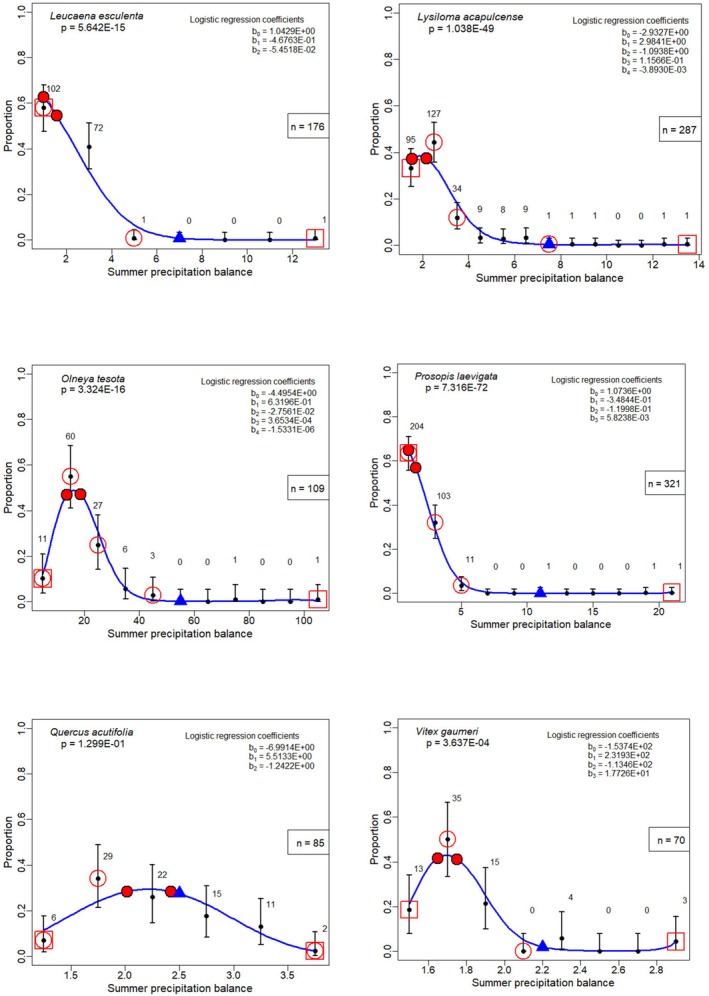
Occurrences along an environmental gradient of **summer precipitation balance** (continued).

**FIGURE D29:**
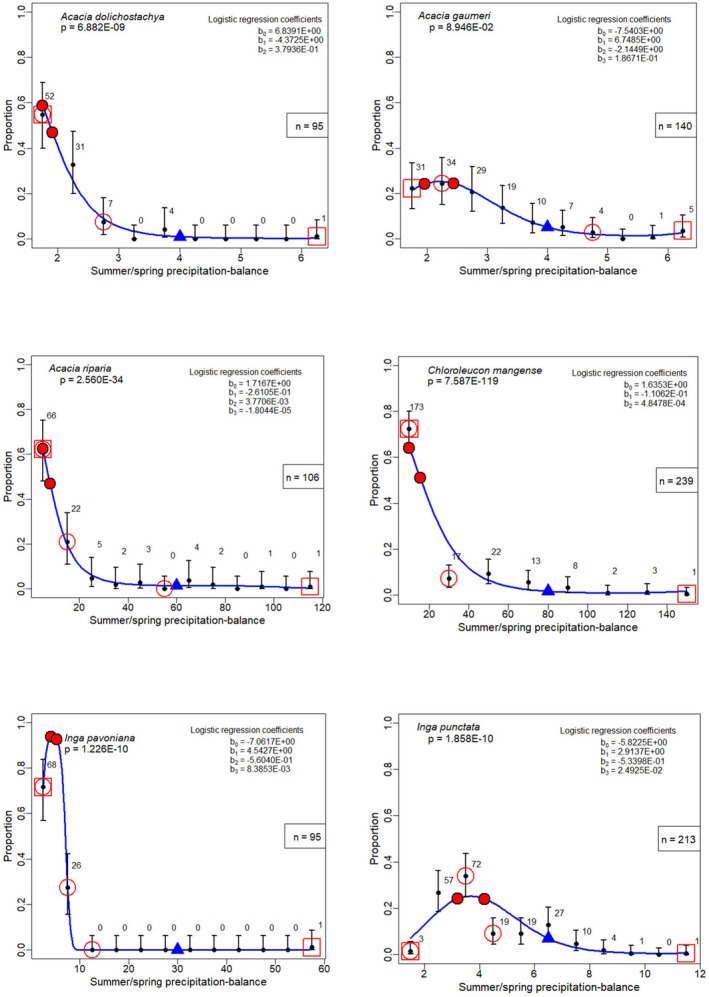
Occurrences along an environmental gradient of **summer/spring precipitation‐balance**.

**FIGURE D30:**
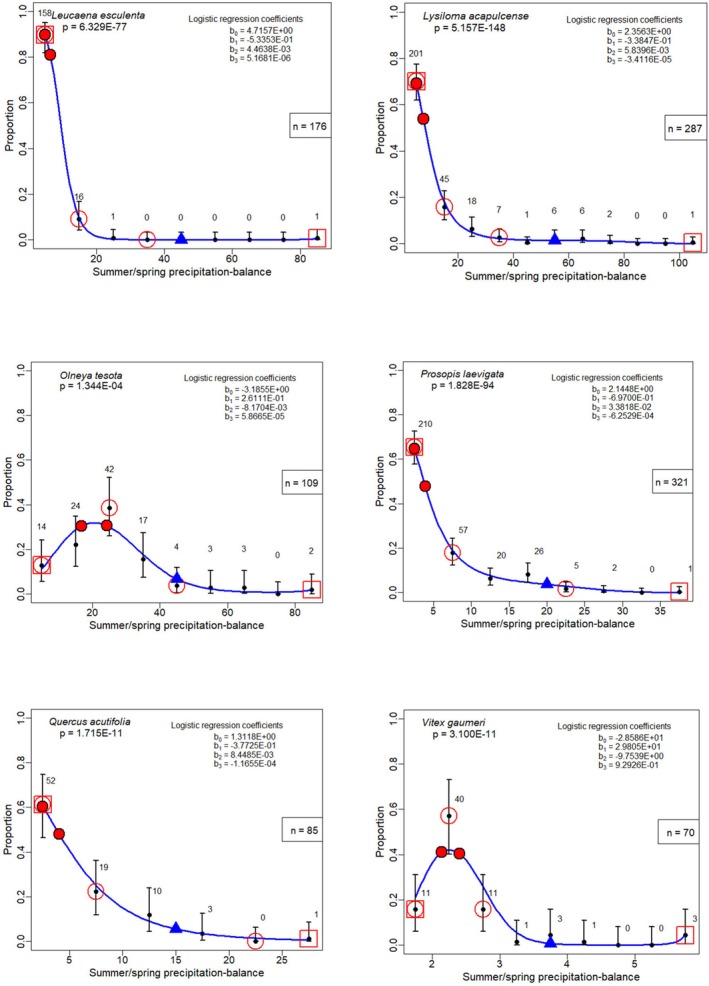
Occurrences along an environmental gradient of **summer/spring precipitation‐balance** (continued).

**FIGURE D31:**
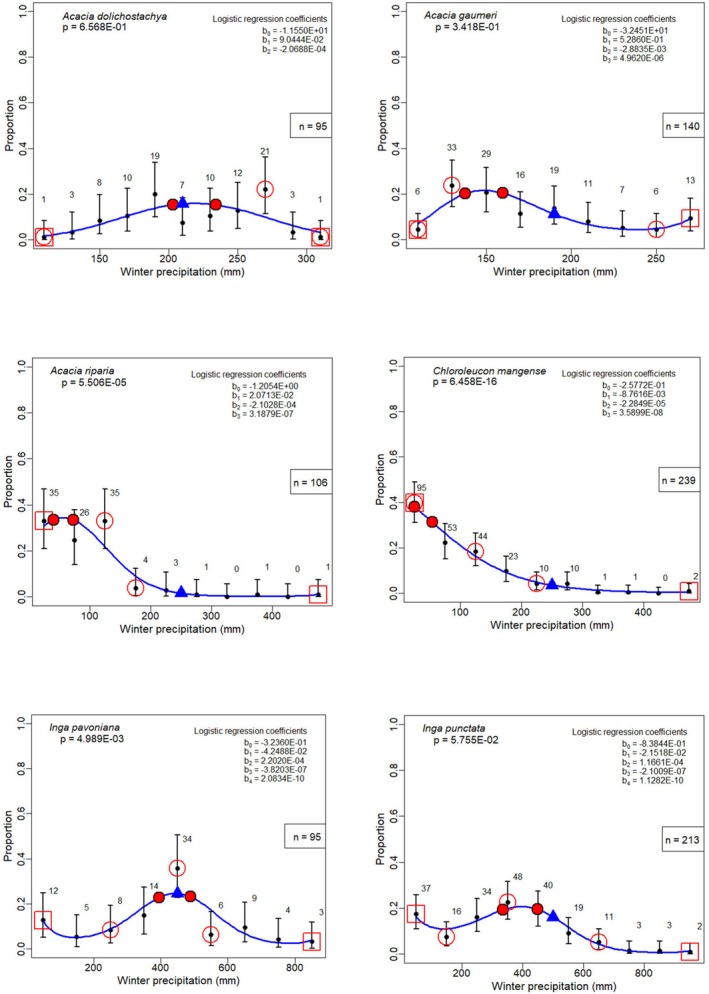
Occurrences along an environmental gradient of **winter precipitation**.

**FIGURE D32:**
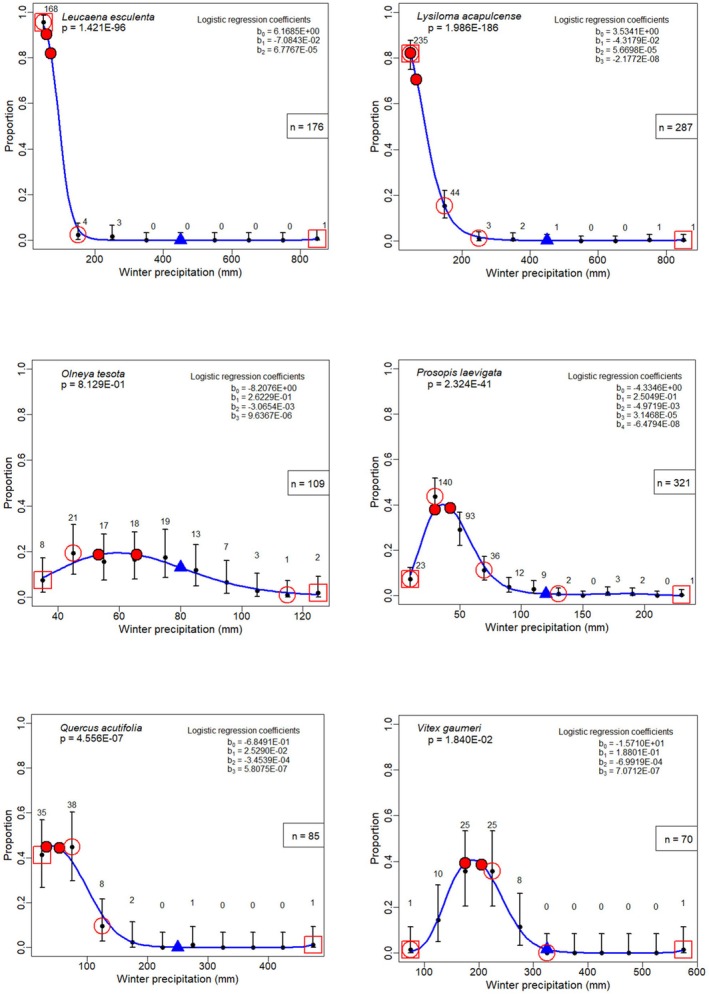
Occurrences along an environmental gradient of **winter precipitation** (continued).

## Data Availability

All data are available as Supporting Information in three Excel sheets (S1a to S1c) at https://doi.org/10.17632/3pyh9jpgzg.1 (Antúnez and Ricker 2025):
S1a: Environmental raw data for each species (XLSX, first sheet);
S1b: Parameters for all species‐variable regressions (XLSX, second sheet);
S1c: Required limits for symmetrical tails around the midpoint (XLSX, third sheet).

## Endnotes

^1^
The reason is that a standard significance test between two estimated means ${\hat{M}}_{1}$ and ${\hat{M}}_{2},$ that follow a normal distribution (here according to the central limit theorem), corresponds to calculating, if the interval $\left({\hat{M}}_{1}-{\hat{M}}_{2}\right)\pm 1.96\cdot \sqrt{{\hat{\mathrm{SE}}}_{1}^{2}+{\hat{\mathrm{SE}}}_{2}^{2}}$ includes zero. On the other hand, comparing two confidence intervals corresponds to observe if ${\hat{M}}_{1}\pm 1.96\cdot {\hat{\mathrm{SE}}}_{1}$ and ${\hat{M}}_{2}\pm 1.96\cdot {\hat{\mathrm{SE}}}_{2}$ overlap. The overlap method is always less powerful to detect different means, because the sum of the two half‐intervals is larger than the confidence interval: $1.96\cdot {\hat{\mathrm{SE}}}_{1}+1.96\cdot {\hat{\mathrm{SE}}}_{2}\geq 1.96\cdot \sqrt{{\hat{\mathrm{SE}}}_{1}^{2}+{\hat{\mathrm{SE}}}_{2}^{2}}$ converts to ${\hat{\mathrm{SE}}}_{1}\cdot {\hat{\mathrm{SE}}}_{2}\geq 0,$ which is always true.

## Appendix A

### Distribution Maps for the 12 Species in Mexico

Note: Collection points are shown for each species in Mexico. *Acacia gaumeri* is the only species that is endemic in Mexico.

## Appendix B

### Summary Table of Environmental Variables

**Table:**

Nr	Variable	Explanation	Range
1	Annual dryness index	Degree days	1.5–81.2
2	Average slope of the terrain (°)	Average slope of the terrain (site) in degrees	0°–62.5°
3	Cation exchange capacity of soil (cmolc/kg)	A measure of how many cations can be retained on soil particle surfaces, here at a depth of 30–60 cm	8–58 cmolc/kg
4	Elevation above sea level (m)	Vertical distance in meters between sea level and the site's level	0–2794 m
5	Mean annual precipitation (mm)	Total precipitation over a year, and averaged from 1961 to 1990	72–4390 mm
6	Mean annual temperature (°C)	The sum of the daily mean temperature over the year divided by 365, and averaged from 1961 to 1990	13.0°C–29.5°C
7	Mean temperature in the coldest month (°C)	The coldest month is determined from daily mean temperature, and averaged for each month from 1961 to 1990; the resulting temperature is taken	10.1°C–27.0°C
8	Mean temperature in the warmest month (°C)	The warmest month is determined from daily mean temperature, and averaged for each month from 1961 to 1990; the resulting temperature is taken	14.7°C–33.1°C
9	Ratio of summer to total precipitation	“Growing‐season precipitation” divided by the “Mean annual precipitation”	0.27–0.95
10	Soil organic‐carbon content (g/kg)	The carbon content in dead and alive plant parts, animals, and microorganisms in the soil, measured at a depth of 30–60 cm	0.5–93.0 g/kg
11	Soil's pH value	The acidity or basicity of an aqueous solution, measured in a slurry of soil mixed with water	5.2–8.5
12	Spring precipitation (mm)	Total precipitation in April and May, averaged from 1961 to 1990	1–721 mm
13	Summer precipitation (mm)	Total precipitation in July and August, averaged from 1961 to 1990	12–1483 mm
14	Summer precipitation balance	Total precipitation in July, August, and September, divided by the total precipitation in April, May, and June, and averaged from 1961 to 1990	0.9–112.0
15	Summer/Spring precipitation‐balance	Summer precipitation divided by spring precipitation	0.8–152.5
16	Winter precipitation (mm)	Total precipitation from November to February, and averaged from 1961 to 1990	7–991 mm

## Appendix C

### Description of the Algorithm to Find the Highest‐Probability Interval

The method is easiest explained by means of the example from Figure 1. The logistic‐regression equation in this case is

(1)
$$p\left(x\right)=\frac{1}{1+\exp\left[-\left(\begin{array}{l} -28.67+11.19\cdot x-1.646\cdot x^{2}+0.1119\cdot x^{3}\\ -0.003661\cdot x^{4}+4.629\cdot 10^{-5}\cdot x^{5} \end{array}\right)\right]}$$

where *x* here is the annual dryness index. There is no analytical formula to solve the indefinite integral, but the corresponding numerical integration, calculated with *Mathematica*'s function “NIntegrate” from 3 to 27, results in

$$\int_{3}^{27}p\left(x\right)=1.9911.$$

This area is shown in Figure 2 in light and dark gray. The quarter area (25%) is 0.4978.

To find numerically the limits of the highest‐probability interval $(x_{L}$ and $x_{U}),$ as shown in Figure 2 for the example of annual dryness index of *Chloroleucon mangense*, first the mode is needed (in some cases, the mode falls on an endpoint, and then is known without calculation). For that purpose, the *x*‐value of the global maximum of the function $p\left(x\right)$ from (1) is determined between the endpoints for *x* (in *Mathematica* with the function “NMaximize”). In the example, the solution s 7.345 between the endpoints 3 and 27.

Since for $p\left(x\right)$ there is no analytical solution of an indefinite integral, a seventh‐degree polynomial $\tilde{p}\left(x\right)$ is determined as a sufficiently accurate substitute function of (1). To determine the coefficients of the polynomial, 101 data points of the original regression function in a short interval around the mode (in this case between x=5 and 10) are calculated, and the *Mathematica* function “FindFit” is applied; a curvilinear regression could also be used directly for this purpose. Here, the result is

(2)
$$\begin{array}{c} \tilde{p}\left(x\right)=76.97-70.28\cdot x+27.11\cdot x^{2}-5.758\cdot x^{3}+0.7322\cdot x^{4}\\ -0.05595\cdot x^{5}+0.002381\cdot x^{6}-4.354\cdot 10^{-5}\cdot x^{7}. \end{array}$$

The indefinite integral for a seventh‐degree polynomial is

$$\begin{array}{cc} \int_{x_{L}}^{x_{U}}\tilde{p}\left(x\right)\;\mathrm{dx}= & b_{0}\cdot \left(x_{U}-x_{L}\right)+\frac{b_{1}}{2}\cdot \left(x_{U}^{2}-x_{L}^{2}\right)+\frac{b_{2}}{3}\cdot \left(x_{U}^{3}-x_{L}^{3}\right)\\ & +\frac{b_{3}}{4}\cdot \left(x_{U}^{4}-x_{L}^{4}\right)+\frac{b_{4}}{5}\cdot \left(x_{U}^{5}-x_{L}^{5}\right)+\frac{b_{5}}{6}\cdot \left(x_{U}^{6}-x_{L}^{6}\right)\\ & +\frac{b_{6}}{7}\cdot \left(x_{U}^{7}-x_{L}^{7}\right)+\frac{b_{7}}{8}\cdot \left(x_{U}^{8}-x_{L}^{8}\right), \end{array}$$

and with the coefficients from (2):

(3)

We wish to find $x_{L}$ and $x_{U}$ around the mode, such that

$$\int_{x_{L}}^{x_{U}}\tilde{p}\left(x\right)\;\mathrm{dx}=\frac{1}{4}\cdot 1.9911=0.4978.$$

Half of the area is to the left of the mode, and the other half of the area to the right (Figure 2). Therefore, one can divide the area into two half‐areas of 0.4978/2=0.24889, and replace $x_{U}$ with $x_{\text{mode}}=7.345$ in (3), to get the following equation:

(4)
$$\begin{array}{l} 73.18-76.97\cdot x_{L}+35.14\cdot x_{L}^{2}-9.036\cdot x_{L}^{3}+1.439\cdot x_{L}^{4}-0.1464\cdot x_{L}^{5}\\ +0.009325\cdot x_{L}^{6}-3.402\cdot 10^{-4}\cdot x_{L}^{7}+5.443\cdot 10^{-6}\cdot x_{L}^{8}=0.24889, \end{array}$$

whose solution is $x_{L}=6.64$. The solution of (4) is a polynomial root that can be solved in *Mathematica* with “NSolve”. Subsequently, $x_{U}$ was calculated with half the area left to the mode:

$$\begin{array}{l} -73.18+76.97\cdot x_{U}-35.14\cdot x_{U}^{2}+9.036\cdot x_{U}^{3}-1.439\cdot x_{U}^{4}+0.1464\cdot x_{U}^{5}\\ -0.009325\cdot x_{U}^{6}+3.402\cdot 10^{-4}\cdot x_{U}^{7}-5.443\cdot 10^{-6}\cdot x_{U}^{8}=0.24889, \end{array}$$

which yields $x_{U}=8.05$. The corresponding function values are calculated with (1). In cases, where the mode is on an endpoint, the whole quarter area is calculated to one side of the mode. In Figure 2, the area of the highest‐probability interval is shown in dark gray. The calculated parameters are here $x_{L}=6.64$, $x_{\text{mode}}=7.345$, and $x_{U}=8.05$, and the function values are $f\left(x_{L}\right)=0.337,f\left(x_{\text{mode}}\right)=0.360$, and $f\left(x_{U}\right)=0.341$.

## Appendix D

### Regression graphs

The graphs in the following Figures D1, D2, D3, D4, D5, D6, D7, D8, D9, D10, D11, D12, D13, D14, D15, D16, D17, D18, D19, D20, D21, D22, D23, D24, D25, D26, D27, D28, D29, D30, D31, D32 are ordered first according to the environmental variable, and subsequently according to species. As in Figure 3, each graph includes the species name, the environmental variable, the regression coefficients, the total number of data points (“*n*”), and the probability (“*p*”) of a chi‐squared test for differences among the proportions that were calculated from the detected frequencies of tree occurrences. The proportions can be interpreted as probabilities to find the tree species at that point of the environmental gradient. The *p*‐value under the species name refers to the probability of a chi‐squared test to detect differences among the proportions (represented by black circles) according to Fleiss (2003: 188–189). The numbers above the bars show the frequencies of collection points at that environmental parameter, and “*n*” refers to the total number (sum) of all collection points, with which the proportions were calculated. The blue triangle represents the midpoint of the environmental variable. The red filled circles give the limits of the parameters (on the *x*‐axis), within which individuals are found with 25% probability, as derived from the regression result. The red squares and circles that are not filled, served to determine the degree of the polynomial regression equation, as explained in the article's text.
